# Teacher pay in Africa: Evidence from 15 countries

**DOI:** 10.1016/j.worlddev.2022.105893

**Published:** 2022-07

**Authors:** David K. Evans, Fei Yuan, Deon Filmer

**Affiliations:** a Center for Global Development, USA; b Harvard Graduate School of Education, USA; c World Bank, USA

**Keywords:** Education, Teachers, Public sector, Teacher earnings

## Abstract

Pay levels for public sector workers—and especially teachers—are a constant source of controversy. In many countries in Sub-Saharan Africa, protests and strikes suggest that pay is low, while comparisons to average national income per capita suggest that it is high. This study presents data on teacher earnings from 15 African countries. The results suggest that in several (seven) countries, teachers’ monthly earnings are lower than other formal sector workers with comparable levels of education and experience. However, in all of those countries, teachers report working significantly fewer hours than other workers, such that hourly earnings are significantly lower for teachers in only one country. The study documents non-pecuniary benefits (such as medical insurance or a pension) for teachers relative to other workers: of the 13 country surveys that report non-pecuniary benefits, teachers are more likely to receive at least one benefit than other workers in 11. Teachers who report fewer hours are no more likely to report holding a second job, although teachers overall are nearly two times more likely to hold a second job than other workers. The study documents other characteristics of the teacher labor force across countries—e.g., mostly male but less so than other workers, mostly employed by the public sector. The study also documents within-country variation across types of teacher contracts—e.g., teachers on fixed term contracts make about 70 percent of teachers on permanent contracts, with wide variation across countries. The large heterogeneity in teacher earnings premia is not easily explained by observed characteristics of the countries’ economies or education systems. Nonetheless, after taking hours and non-pecuniary benefits into account, we find no evidence that teachers are systematically underpaid in this sample of countries.

## Highlights

• We examine micro-data on teacher pay and on pay for other workers in 15 African countries.
• In seven countries, teachers’ monthly earnings are lower than other formal sector workers with comparable levels of education and experience.
• In all seven of those countries, teachers report working significantly fewer hours than other workers, such that hourly earnings are significantly lower for teachers in only one country.
• Of the 13 country surveys that report non-pecuniary benefits, teachers are more likely to receive at least one benefit than other workers in 11.
• Teachers are nearly two times more likely to hold a second job than other workers.

## 1 Introduction

Determining the right level and structure for public sector salaries is a challenge around the world. Nowhere is this more salient than among teachers. The last few years have seen teacher strikes across the African continent, with salary often at the center of demands.^1^ Yet evidence on the efficacy of raising teacher salaries for boosting student learning outcomes is mixed, with cross-country analysis showing that countries with higher teacher salaries tend to have higher student learning outcomes but individual country experiences showing little impact of blanket raises in teacher pay (de Ree et al., 2018, Hanushek et al., 2019). These current events, along with recent findings in the literature, raise a variety of questions about teachers and teacher compensation: Who makes up the teaching workforce? How has the supply of teachers changed over time? What do teachers and other workers earn? How are teachers paid relative to other professionals? How do teacher earnings differentials vary with their reported hours worked, their non-pecuniary benefits, and their contract type? Do aspects of the economy or of the education system explain the heterogeneity in teacher earnings differentials across countries? In this paper, we seek to establish a series of descriptive patterns around the levels of teacher earnings and demonstrate where there are regularities (or not) across 15 Sub-Saharan African countries.^2^

The challenge in Africa is particularly pressing. In order to achieve universal primary and secondary education, Sub-Saharan African countries are predicted to need 17 million additional teachers (UNESCO, 2016). The quality of the incoming teachers will significantly affect the educational outcomes of children in African countries, as teacher quality is a critical determinant of students’ test scores, non-test score behaviors, and long-term outcomes (Araujo et al., 2016, Chetty et al., 2014, Hanushek and Rivkin, 2010, Jackson, 2012). Furthermore, these teachers will join education systems characterized by poor performance (Bold et al., 2017). Pay levels may draw more candidates into the teaching profession, may affect the quality of candidates drawn to the profession, and may affect turnover within the profession (Okafor, Iriobe, Nwajiaku, & Onyene, 2016).

The challenge is compounded by the fact that there is limited documentation of this policy-relevant topic in Sub-Saharan Africa, in part because there are no systematic teacher salary data across African countries. UNESCO occasionally documents teacher salaries in terms of country GDP per capita (e.g., UNESCO, 2009),^3^ but it does not provide information on relative earnings differentials. Understanding the structure of earnings in countries with similar economic structures may be one input into policymaker discussions about setting salaries.

In this study, we assemble data from representative population or labor force surveys across 15 Sub-Saharan African nations. We characterize the demographics of the teaching workforce, compare earnings from the primary job for teachers to other wage workers of similar age, education (focusing on both teachers and other workers with at least secondary education), rural/urban sector, and gender. We explore how earnings premia vary with hours worked and the potential role of non-pecuniary benefits. We then explore factors that may explain heterogeneity in teacher wage differentials.

Our analysis shows that, across countries, teacher earnings rise in absolute terms with GDP per capita but that they fall as a percentage of GDP per capita – i.e., they rise more slowly than GDP per capita. Within countries, we find that in seven out of the 15 countries, teachers’ monthly earnings are statistically significantly lower than those of other comparable wage workers. In five countries (Burkina Faso, Côte d’Ivoire, Namibia, Senegal and Zambia) teachers’ monthly earnings are statistically significantly higher than comparable workers. But simple earnings comparisons are complicated by adjustments for working hours. In four of the seven countries where teachers’ monthly earnings are lower and where teachers report their hours, their hourly wage is not statistically different than that of other workers (and point estimates are higher for teachers). Once adjusted for hours, in only one country (Nigeria) are teacher earnings significantly lower than those for other workers. Furthermore, teachers report a higher likelihood of receiving non-pecuniary benefits than other workers in most countries in our sample. While we cannot quantify the value of those non-pecuniary benefits, they suggest that—once accounting for hours and benefits—teachers are not systematically underpaid.

Positive premia tend to be higher for teachers on permanent contracts—who get paid more but work similar hours—than for teachers on fixed-term contracts. Teacher earnings are not more determined by age and education than those of other workers. The fraction of teachers in the workforce has risen across countries over the last two decades. Despite the high degree of heterogeneity in teacher earnings premia across countries, neither characteristics of the economy (GDP per capita, the share of wage work in the labor force, or female labor force participation) nor characteristics of the education system (teacher unionization, the role of the private sector in education, the growth in the teacher labor force, etc.) are highly associated with those premia. This suggests that for the most part, the heterogeneity is driven by idiosyncratic country histories and characteristics.

Our analysis contributes to the literature on cross-country differences in teacher salaries. Finan, Olken, and Pande (2017) document that low-income countries provide higher wage premia to public sector employees. Mizala and Ñopo (2016) conduct an analysis of teacher relative pay using data from 13 Latin American countries and find that teachers earn less than “other professionals and technicians” with similar observable characteristics. Our analysis on low- and middle-income countries in Sub-Saharan Africa finds that earnings premia are higher in countries with higher incomes, but we also observe substantial variation in teacher earnings premia given the income level.^4^

The analysis in this study also relates to the literature on attracting quality candidates to the public sector and, in the case of education, the subsequent impacts on students. Ferraz and Finan (2009) document that higher wages for politicians improve political performance in Brazil. In addition, Dal Bó, Finan, and Rossi (2013) find that when salaries increased, more able applicants applied for public-sector jobs. With respect to teachers, combining cross-country data on teacher salary and student performance on international assessments in OECD countries, Dolton, Marcenaro-Gutierrez, Pistaferri, and Algan (2011) suggest that “the relative wage of teachers is a very good proxy for their average quality.”

One major reason that education systems care about teacher pay is because of its potential impacts on student outcomes. While our study stops short of examining the impact of teacher salaries on student learning or other student outcomes, a careful understanding of teacher pay is crucial to the subsequent question of how it impacts students. The literature is mixed on this. On the one hand, simple pay increases had no impact on student learning in Indonesia (de Ree et al., 2018). On the other hand, several studies examine the effect of providing hardship allowance to teachers working in remote areas in low- and middle-income countries on student performance, and most studies that report measures of student learning find positive impacts (Evans & Mendez Acosta, 2021a). Hanushek et al. (2019) show that in OECD countries, teacher salary premiums are associated with both teacher cognitive skills and student performance. Ultimately, the relationship between teacher pay and student learning may operate through multiple mechanisms (e.g., effort, quality of candidates, and turnover) and may vary between the short- and long-run. By characterizing teacher earnings across countries, our analysis provides building blocks for future work on the quality of teachers and teaching in Sub-Saharan Africa.

## 2 Data, analytical strategy, and empirical strategy

### 2.1 Data

We draw on several sources of data. Our main analysis of teachers and their earnings is based on household and labor force surveys conducted in 15 low- and middle-income countries in Sub-Saharan Africa (Table 1).^5^ We chose these countries and surveys based on three criteria. First, the surveys included detailed information on occupation, working hours, earnings, educational attainment and other basic demographic characteristics of surveyed household members or individuals. Second, the surveys were conducted no earlier than 2010. If there were multiple surveys available for the same country, we chose the most recent one. This allows us to provide a contemporary characterization of teacher pay across several African countries, especially when describing current levels and comparing those to other professions. Third, the survey data were accessible.

**Table 1:** Description of analyzed datasets.

			Teachers		
Country	Source	Year	Number surveyed	Percent of total surveyed wage workers	Percent of total surveyed work force (ages 15–64)
		(1)	(2)	(3)	(4)
Burkina Faso	Continuous Multisectoral Survey	2014	343	13%	1.3%
Côte d'Ivoire	National Employment Survey	2013	201	6%	1.1%
Democratic Republic of Congo	National Household Survey	2012	1,104	18%	3.1%
The Gambia	Integrated Household Survey	2010	243	10%	1.9%
Ghana	Ghana Living Standards Survey 6	2012–2013	772	16%	2.4%
Liberia	Household Income and Expenditure Survey	2014–2015	171	14%	2.2%
Malawi	Integrated Household Panel Survey	2010	393	12%	1.9%
Namibia	Labor Force Survey	2013	359	8%	3.7%
Niger	National Survey on Household Living Conditions and Agriculture	2014	119	12%	1.6%
Nigeria	General Household Survey	2015	240	20%	2.8%
Senegal	Poverty Monitoring Survey	2010	331	7%	0.8%
Sierra Leone	Labor Force Survey	2014	220	27%	3.1%
Tanzania	Labor Force Survey	2014	396	12%	1.4%
Uganda	Living Standards Measurement Survey	2013	170	15%	2.6%
Zambia	Labor Force Survey	2014	486	10%	2.4%

*Source:* The surveys are publicly available in the World Bank Microdata Library.

The resulting set of countries includes Côte d’Ivoire, Namibia, Sierra Leone, Tanzania and Zambia with data from national labor force surveys; Burkina Faso, Malawi, Niger, Nigeria and Uganda with data from Living Standards Measurement Surveys (LSMS) that include an employment module; and the Democratic Republic of Congo, The Gambia, Ghana, Liberia and Senegal with data from national household surveys that include a labor force module. Table 1 provides background information on these surveys: the countries, the source of the survey data and the year in which the survey was conducted.

The focus of our analysis is primary and secondary school teachers. This choice was driven by the fact that teachers in the basic education cycle make up the bulk of the teaching workforce. Additionally, their salaries are often set centrally and typically paid directly out of government expenditure on education, which makes cross-country comparison possible and meaningful. From each of these surveys, we identified primary and secondary teachers using occupation codes listed in interviewer manuals. Most of the surveys used the International Standard Classification of Occupations (ISCO) or a variant form of it. The occupation codes allowed us to differentiate primary and secondary teachers from other educators such as pre-primary teachers, special education teachers, and university lecturers and professors. Exceptions are Malawi where the data classifies teachers at all levels in the same group. (Detailed data processing notes can be found in Appendix Table A1.) We retained Malawi in our analysis, but our conclusions are similar if we exclude it. We excluded educators other than primary and secondary teachers from our analytical samples since their jobs and pay determination may be quite different from primary and secondary teachers. Throughout this paper, “teachers” therefore only refers to primary and secondary school teachers.

Table 1 provides details about our samples: the number of teachers, the share of teachers in the formal sector, and the shares of teachers in the overall workforce in each survey. The number of teachers sampled ranges from 119 teachers in Niger to 1,104 teachers in the Democratic Republic of Congo, with 370 teachers on average per country. On average, teachers in these countries represent 2.2 percent of the overall labor force in each survey.^6^ This share is comparable to that in high-income countries like the United States, where primary and secondary teachers make up about 2.2 percent of the labor force.^7^ Across the surveys, teachers on average constitute 13 percent of wage employment.^8^

We also include supplementary analysis using data from a teacher survey administered in francophone African countries in 2014, the PASEC (PASEC, 2015).^9^ We include data from all available countries: Benin, Burkina Faso, Burundi, Cameroon, the Democratic Republic of Congo, Côte d'Ivoire, Niger, Senegal, Chad, and Togo. Finally, we present data on the total numbers of teachers and students from the UNESCO Institute for Statistics (UNESCO, 2020) and data on the labor force across countries from the International Labour Organization (ILO, 2020).

### 2.2 Analytical strategy

This paper takes an exploratory approach to questions around teacher pay in Sub-Saharan Africa. Rather than establishing a theoretical framework—which has been done for teachers (Crawfurd & Pugatch, 2020) and for public sector workers more broadly (Finan et al., 2017)—we interrogate the data with the aim of establishing a series of stylized facts and exploring where there are empirical regularities across countries (and where there are not). Specifically, we structure our analysis around five main questions that we hope will help to motivate future work on teachers.

*Question 1: Who makes up the teaching workforce?* This descriptive section explores the demographics of the teaching workforce. We explore various aspects, including gender and age. In some regions of the world, teaching was historically one of the few professions that women could access, although that has changed as other labor market opportunities for women have expanded (Elacqua, Hincapié, & Mariana Alfonso, 2018). The age of the workforce has implications for how quickly one could expect to observe changes over time. We also characterize the proportion of teachers in public versus private schools (since that distribution affects the workings of the teacher labor market) and the distribution of teachers on permanent versus fixed contracts (which also has implications for the incentives of teachers). Finally, we explore the educational attainment of teachers. While additional years of education may or may not affect teaching quality (depending on the quality of that education), it may affect competition across professions.

*Question 2: How has the supply of teachers changed over time?* Many African countries have dramatically expanded access to education in recent decades (Evans & Mendez Acosta, 2021b). This means that the demand for teachers has risen markedly, which has significant implications both for the level of compensation (needing to attract individuals to the teaching profession) and the share of public resources made up by teacher salaries.

*Question 3: What do teachers and other workers earn?* We summarize teacher earnings in different ways that teachers and other workers may conceptualize those earnings: monthly versus hourly and in the context of whether or not they have a second job.^10^ Teachers are seeking to optimize income and other outcomes throughout their full range of economic activities, not just their teaching job.

*Question 4: How are teachers paid relative to other professionals?* Teacher earnings relative to other professionals has implications for the ability of the teaching profession to attract candidates as well as broader perceptions of the fairness of the pay scale (which could, for example, affect the likelihood of teacher strikes). We do not present a causal model of teacher pay: individuals select into the teaching profession for reasons that are not captured in our data. However, we do control for a range of observable characteristics, as discussed below. While teaching is not a purely competitive market (since so much of the market is dominated by the public sector), there are competitive elements in that candidates’ occupational choice may in part be determined by relative earnings, along with other factors such as job security.

*Question 5: How do teacher earnings differentials vary with their reported hours worked, their non-pecuniary benefits, and their contract type?* Although workers may principally consider earnings in terms of monthly or weekly paychecks, differential hours mean that teachers or other workers may have different earnings per hour. We adjust earnings based on the hours reported by workers in the survey. We report data on non-pecuniary benefits (such as pensions or medical insurance) for teachers and other workers. While we have insufficient data to quantify the value of these benefits, we discuss qualitatively how they would likely affect our estimates. Finally, we document the high variation in teacher earnings within countries across fixed-term versus permanent contracts.

*Question 6: Do aspects of the economy or of the education system explain the heterogeneity in teacher earnings differentials across countries?* We observe large variation in teacher earnings premia across countries. To understand the sources of this variation, we document aspects of the economy and the education system that we hypothesize could explain the variation and present correlations between each of these and teacher earnings premia.

We propose that all six of these questions have implications for policy and for future research as scholars identify ways to better identify causal relationships (or the lack thereof) underlying the associations that we report. Furthermore, we propose that a multi-country approach can help researchers and policy makers to avoid the temptation to generalize from just one or two individual country studies.

### 2.3 Empirical strategy for estimating relative earnings

Most of our results are cross-tabulations of the data presented to give the reader a series of descriptive statistics about the teaching profession across the 15 Sub-Saharan African countries we study. Simply presenting descriptive statistics—stratified by certain characteristics—can shed light on general patterns but may not capture systematic differences associated with occupations. For example, there might be different job requirements for teachers, and observed differentials may pick up the effects of age or experience. Additionally, there may be regional differences; for instance, urban or rural residence might be systematically related to both earnings as well as the probability of being a teacher. Thus, in this analysis, besides providing descriptive statistics of teachers and their compensations, we also conduct a set of multivariate analyses to examine teacher earnings differentials. Specifically, we estimate models that control for basic productivity factors (following Gregory & Borland, 1999), as well as other potential confounders—namely gender and urban/rural residence—that might differ systematically between teachers and comparable wage workers.

We use the specification in Equation (1) to run a set of multivariate OLS regressions to estimate earnings differentials that control for observed potentially confounding variables:

(1) $$\ln{(Earning}_{i})=\gamma_{0}+\gamma_{1}{\mathrm{Teacher}}_{i}+\gamma_{2}{Post\_secondary}_{i}+\gamma_{3}{\mathrm{Age}}_{i}+\gamma_{4}{\mathrm{Age}}_{i}^{2}+\gamma_{5}{\mathrm{Male}}_{i}+\gamma_{6}{\mathrm{Urban}}_{i}+\varepsilon_{i}$$

where ln*(Earning)* is the log of total reported earnings (monthly or hourly, depending on the specification), *Teacher* is an indicator variable for teachers, *Post-secondary* is an indicator for having post-secondary education, *Age* is the individual’s age, *Male* is an indicator for male individuals, and *Urban* is an indicator for urban residents. In order to further ensure that we are comparing teachers to comparable workers, the regression sample is limited to wage workers with secondary or post-secondary education.^11^

Even though we control for several potentially confounding observed variables, the estimated coefficient may still not represent the isolated impact of being a teacher on earnings, as there may be unobserved factors that affect both earnings and the probability of being teacher. If this is the case, the coefficient on the teacher variable would capture both the actual impact of being a teacher and the impact of these unobserved factors. Therefore, the analysis should be viewed simply as a summary of the observed premium (or deficit) of being a teacher controlling for the variables included in the model.

When calculating total earnings, we combine reported payments in cash as well as payments in kind (if any), such as food and allowances.^12^ However, five of the 15 countries in our sample (Côte d’Ivoire, the Democratic Republic of Congo, The Gambia, Namibia and Tanzania) did not explicitly report the existence and value of in-kind payments, and one country (Senegal) explicitly asked respondents to include in-kind payments in their total earnings (Appendix Table A2, column 2). It is unclear from the questionnaires whether in these cases in-kind payments are included in the reported values or not.^13^ If in-kind payments are included in these cases, our measures of earnings can be interpreted as the total earnings of an employed person from their primary job. However, teachers who are civil servants receive further benefits that are not likely to be monetized in regular earnings (e.g., retirement benefits; job security) and hence not included in the response to the earnings questions. We explore the degree to which teachers and other workers report these non-pecuniary benefits and the implications for total teacher compensation relative to other workers in results section 3.4.3.

In our analysis, we separately consider monthly and hourly earnings since hours worked differ systematically between wage workers who are teachers and those who are not. For all but one survey we analyze, earnings are reported along with the corresponding time unit of the earnings, for example, daily, weekly, monthly, quarterly, or yearly. Using these two indicators, we first derive implied monthly earnings. For example, if an employee reports her quarterly earnings, then her monthly earnings are calculated as the quarterly earnings divided by three. If daily earnings are reported, we multiply the daily earnings by how many days the person usually works in a week, then multiply that number by four to get monthly earnings. To estimate hourly earnings, we divide the monthly earnings by four times the reported weekly working hours for each worker. When daily working hours are reported, we calculate the weekly working hours by multiplying the number of days worked in a week. The survey from The Gambia did not ask about working hours, so we exclude it from any analysis involving hourly earnings. (Detailed data processing notes can be found in Appendix Table A1.).

When reporting earnings and performing the multivariate analysis, we limit the sample to wage employees (i.e., who self-identified as a paid employee) with at least some secondary education to establish a group of professionals comparable to teachers.^14^ This is driven by two facts. First, on average only 3.4 percent of teachers had less than secondary education in the countries in our sample (Table 2). Therefore, we assume that secondary education is the minimum education requirement for being a teacher in most cases and exclude observations with less than that. Second, teachers in both public and private schools are wage workers. The earnings profiles of wage workers could be systematically different from workers in the informal sector. For instance, many workers in the informal sector are seasonal, so their earnings would fluctuate substantially. A large share of workers in the countries in our sample were either self-employed farmers or family helpers who did not report their earnings. Additionally, as a robustness check to allow for outliers we also estimate multivariate models that trim the top and bottom 1 percent of monthly or hourly earnings in each country sample, as well as estimate the models using median (rather than OLS) regressions.^15^

**Table 2:** Educational attainment of teachers and other wage workers (proportion of workers).

	Teachers			Other wage workers			Primary teachers			Secondary teachers		
Country	P	S	P-S	P	S	P-S	P	S	P-S	P	S	P-S
	(1)	(2)	(3)	(4)	(5)	(6)	(7)	(8)	(9)	(10)	(11)	(12)
Burkina Faso	6.1	73.1	20.8	59.3	31.8	8.9	7.1	82.7	10.2	0.0	12.8	87.2
Côte d'Ivoire	1.0	54.3	44.7	71.1	21.8	7.1	1.6	78.9	19.5	0.0	13.5	86.5
DR Congo	2.5	82.7	14.8	17.0	56.4	26.6	2.6	86.6	10.8	1.8	62.0	36.3
The Gambia	5.8	36.7	57.5	41.5	47.2	11.4	9.8	42.7	47.6	0.0	27.8	72.2
Ghana	0.4	26.0	73.5	22.5	54.5	23.0	0.5	35.8	63.7	0.3	13.0	86.7
Liberia	1.9	76.9	21.2	39.7	49.8	10.5	2.3	85.2	12.5	0.0	39.3	60.7
Malawi	5.1	65.1	29.8	52.9	36.4	10.7	N.A.			N.A.		
Namibia	1.1	30.1	68.8	18.4	68.0	13.6	0.5	39.0	60.5	2.2	15.4	82.4
Niger	4.2	67.2	28.6	61.4	27.8	10.8	5.4	79.6	15.1	0.0	23.1	76.9
Nigeria	1.7	10.8	87.5	18.5	40.1	41.4	1.9	15.5	82.6	1.3	1.3	97.5
Senegal	2.2	48.5	49.4	57.3	33.6	9.2	3.4	75.3	21.4	0.7	15.8	83.6
Sierra Leone	1.8	22.5	75.7	16.6	50.9	32.5	2.3	26.2	71.5	1.1	17.1	81.8
Tanzania	0.3	34.9	64.9	39.6	36.8	23.6	0.4	44.2	55.4	0.0	12.7	87.3
Uganda	0.6	7.2	92.2	54.4	26.3	19.3	0.7	7.9	91.4	0.0	3.9	96.2
Zambia	15.8	64.8	19.4	17.4	68.9	13.6	0.9	30.2	68.9	0.0	23.1	76.9
**Average**	**3.4**	**46.7**	**49.9**	**39.2**	**43.4**	**17.5**	**2.8**	**52.1**	**45.1**	**0.5**	**20.0**	**79.4**

Notes: P stands for primary school education. S stands for secondary school education. P-S stands for post-secondary school education. The number in each cell represents the proportion of workers with each level of education. Teachers include survey respondents who self-identified as a primary or secondary school teacher and a wage worker. Other wage workers include respondents who self-identified as a wage worker in other occupations; university faculty, special education teachers and other teachers were excluded from the group of other wage workers. Primary teachers include respondents who self-identified as a primary school teacher and a wage worker. Secondary teachers include respondents who self-identified as a secondary school teacher and a wage worker. The survey in Malawi did not differentiate the levels of teachers. *Source:* Surveys listed in Table 1.

To further unpack the earnings differentials for teachers at different education levels, we extended the teacher variable in equation (1) to primary teacher and secondary teacher variables as reflected in equation (2). Again, we fit equation (2) using both OLS and median regressions.

(2) $$\ln\left({\mathrm{Earning}}_{i}\right)=\gamma_{0}+\gamma_{1}{T\_Primary}_{i}+\gamma_{2}{T\_Secondary}_{i}{+\gamma }_{3}{\mathrm{Age}}_{i}+{\gamma_{4}Age}_{i}^{2}+\gamma_{5}{\mathrm{Male}}_{i}+\gamma_{6}{\mathrm{Urban}}_{i}+\varepsilon_{i}$$

where *T_Primary* is an indicator for primary school teachers and *T_Secondary* is an indicator for secondary school teachers.

## 3 Results

### 3.1 Who are teachers in Africa?

Before turning to the regression results, we first document the demographic characteristics of teachers and other wage workers (Table 3). For this analysis, we define teachers as survey respondents who self-identified as a primary or secondary school teacher and a wage worker—which excludes university faculty, special education teachers and other teachers.^16^^,^^17^ A typical teacher in Africa is a 38-year-old male living in an urban area. In absolute terms, teaching is still more likely to be a career for men: on average, three out of five African teachers are male. By way of contrast, three-quarters of teachers are female in the U.S. (NCES, 2018). However, in relative terms, women in the countries in our study are more represented in teaching than in other wage jobs where male workers make up an even larger share (73 percent); these findings are consistent with the pattern in high-income countries like the U.S (U.S. Bureau of Labor Statistics, 2021). Indeed, although we observe a lower share of male teachers in the teaching force as national income levels rise across our sample (corr = -0.58, p-value < 0.05, N = 15), the gender difference between teaching and other wage jobs persists across income levels. For example, in Namibia, the country with the highest GDP per capita in our sample, 35 percent of teachers versus 58 percent of other wage workers are male. In Malawi, one of the low-income countries, while the share of male workers is 60 percent in teaching, the share is 79 percent in other wage jobs.

**Table 3:** Demographic characteristics of teachers in the analytical sample.

	Teachers					Other wage workers		
Country	Age	Percent male	Percent urban	Percent working in private schools	Percent on fixed term/ temporary contracts	Age	Percent male	Percent urban
	(1)	(2)	(3)	(4)	(5)	(6)	(7)	(8)
Burkina Faso	35.1	57.3%	76.6%	20.4%	10.6%	33.0	73.1%	79.6%
Côte d'Ivoire	38.4	79.1%	76.1%	21.0%	41.7%	33.5	75.7%	80.1%
DR Congo	37.5	72.6%	50.6%	15.1%	40.0%	39.7	78.8%	81.2%
The Gambia	35.2	67.5%	67.5%	38.3%	N.A.	33.6	71.7%	79.6%
Ghana	35.0	64.6%	61.4%	17.9%	N.A.	35.9	70.6%	74.7%
Liberia	44.6	85.4%	42.1%	29.4%	N.A.	38.1	80.0%	58.4%
Malawi	38.2	59.5%	41.2%	21.9%	N.A.	34.7	79.2%	47.6%
Namibia	39.6	34.9%	48.7%	4.7%	14.9%	35.9	57.8%	78.2%
Niger	37.1	47.9%	90.6%	18.0%	29.1%	36.2	85.0%	78.2%
Nigeria	40.7	45.4%	45.4%	24.2%	N.A.	39.6	68.1%	54.2%
Senegal	37.1	76.7%	83.4%	9.1%	16.9%	35.6	64.6%	82.0%
Sierra Leone	41.8	63.6%	79.3%	25.5%	16.9%	38.8	69.0%	90.4%
Tanzania	38.6	43.9%	85.4%	19.3%	5.4%	37.5	70.7%	96.3%
Uganda	37.5	60.6%	29.4%	31.2%	40.1%	31.9	71.5%	48.8%
Zambia	35.3	49.6%	60.1%	12.1%	12.6%	33.1	73.1%	69.3%
**Average**	**38.1**	**60.6%**	**62.5%**	**20.5%**	**22.3%**	**35.8**	**72.6%**	**73.2%**

Notes: Teachers include survey respondents who self-identified as a primary or secondary school teacher and a wage worker. Other wage workers include respondents who self-identified as a wage worker in other occupations; university faculty, special education teachers and other teachers were excluded from the group of other wage workers. Teachers were considered working in private schools unless they reported government (at any level) or any public entity as their employer. Surveys in The Gambia, Ghana, Liberia, Malawi, and Nigeria did not report whether a worker was on a permanent or fixed term/temporary contract. *Source:* Surveys listed in Table 1.

The average age of teachers in our study countries ranges from 35 (in Burkina Faso, The Gambia, Ghana, and Zambia) to 45 (in Liberia), with an average of 38. Teachers are about two years older than other wage workers.^18^ About 60 percent of African teachers live in urban areas, which is also where most wage workers are located. Considering that 59 percent of the population of Sub-Saharan Africa lives in rural areas,^19^ the concentration of teachers in urban areas demonstrates a potential mismatch, consistent with findings from previous analysis (Bashir, Lockheed, Ninan, & Tan, 2018).^20^

In terms of school type, about 20 percent of teachers work in private schools in these countries.^21^ This is consistent with the rise of private schools in Sub-Saharan Africa in recent years (World Bank, 2018). But there is significant variation among the countries we study, with private school teachers ranging from 5 percent (in Namibia) to 38 percent (in The Gambia) of the teaching force. Depending on the nature of a private school (e.g., elite or low-cost) and the contract type, teachers’ salary and benefits could vary substantially, which we discuss in section 3.3.

Another important group in the African teaching force is contract teachers. Due to the increasing demand for education, hiring contract teachers is common in Sub-Saharan African countries—as it is in other parts of the world (Chudgar, 2015).^22^ Although their job responsibilities are similar to those of civil-service teachers, contract teachers’ salaries are typically substantially lower, and these teachers lack job security. In the survey data we use for most countries, all employed respondents—including teachers—are asked whether they have a permanent or a fixed-term/temporary contract. The share of teachers on fixed term or temporary contracts ranges from 5 percent (Tanzania) to 42 percent (Côte d'Ivoire) (Table 3). On average, the share of teachers on a fixed term or temporary contract is slightly higher for secondary teachers (25 percent) than for primary teachers (22 percent). In Uganda, the share of teachers on a fixed term or temporary contract at the secondary level (71 percent) is substantially higher than that at the primary level (35 percent).^23^

Table 2 presents the educational attainment of teachers. The majority of teachers have at least secondary school education in all countries in our sample. The only country where more than 7 percent of teachers have only a primary degree is Zambia (15.8 percent). Across countries, about half of teachers have post-secondary education. Relative to other wage workers, teachers are much more likely to have post-secondary education in every country except the Democratic Republic of Congo. There is a notable difference in educational attainment between primary school teachers and secondary school teachers in most of the countries. On average, 80 percent of secondary school teachers have post-secondary education compared to 45 percent among primary school teachers. The difference is less salient in Nigeria and Uganda, where at least 80 percent of both primary and secondary teachers have post-secondary education.

### 3.2 How has the supply of teachers changed over time?

**Fig. 1:**
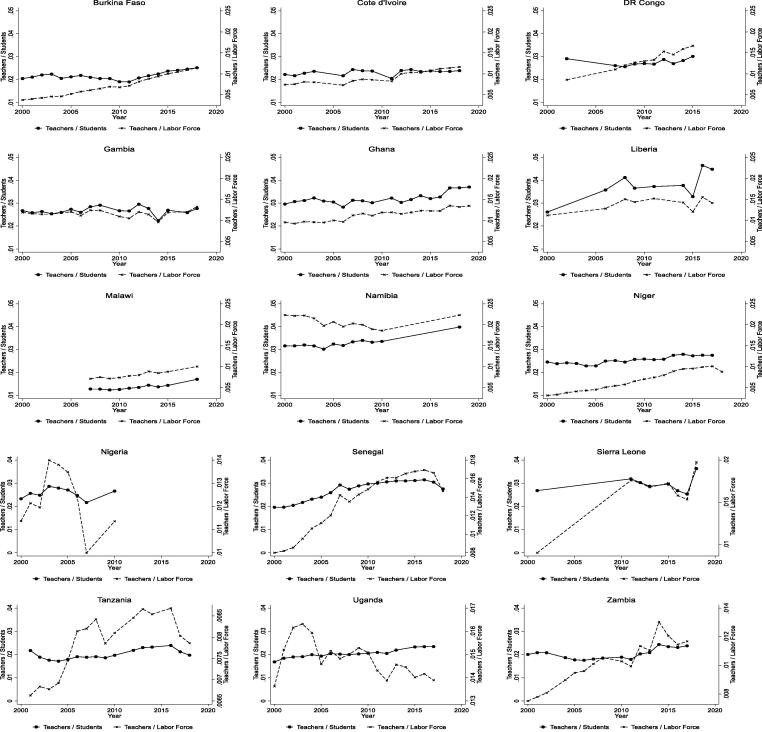
Trends in Primary School Teachers / Students and in Primary School Teachers / Labor Force. Notes: Number of students and number of teachers from UNESCO Institute for Statistics. Labor force is modelled labor force aged 15-64 from International Labor Organization.

Any discussion of teacher earnings makes more sense if we understand the relative supply of workers in the field (which is, in turn, endogenous to teacher earnings). We track the proportion of primary school teachers relative to the overall labor force along with the ratio of primary school teachers to students across the countries in our sample (Fig. 1).^24^ There are no countries in which the fraction of teachers in the labor force has fallen substantively. The proportion has risen sharply in several countries (e.g., Burkina Faso, Democratic Republic of Congo, Sierra Leone, Tanzania, and Zambia), risen modestly in some (e.g., Côte d’Ivoire and Malawi), and stayed roughly constant in others (e.g., The Gambia). Furthermore, the teacher-student ratio has also risen in many countries (i.e., smaller class sizes), so the rise in teachers cannot entirely be explained by rising enrollment. The average rise across the continent suggests a combination of increasing demand for teachers and that teaching is seen as a desirable profession.

### 3.3 What do teachers and other workers earn in Africa?

We assess teachers’ earnings relative to other wage workers in two ways. In this section, we compare monthly earnings, hourly earnings, hours worked weekly and the proportion having a second job (Table 4). In the next section, we report the results of multivariate analyses that simultaneously control for age, gender, and urban/rural residency. For the comparisons of earnings in both this and subsequent sections, we restrict the sample to wage workers with secondary education or more.

**Table 4:** Average working hours and median earnings of teachers and other wage workers.

	Monthly median earnings in PPP ($)		Monthly median earnings relative to GDP per capita		Hourly median earnings in PPP ($)		Hours worked weekly		Proportion of having a second job	
	Teachers	Other workers	Teachers	Other workers	Teachers	Other workers	Teachers	Other workers	Teachers	Other workers
	(1)	(2)	(3)	(4)	(5)	(6)	(7)	(8)	(9)	(10)
Burkina Faso	650	232	4.8	1.7	4.0	1.3	39.2	52.8	22.3%	25.9%
Côte d'Ivoire	848	381	3.4	1.5	5.9	2.1	32.4	46.8	16.0%	12.3%
DR Congo	100	114	1.6	1.8	0.7	0.7	34.3	45.4	46.9%	22.9%
The Gambia	265	235	2.0	1.8	N.A.		N.A.		N.A.	
Ghana	656	389	2.0	1.2	4.4	2.1	34.3	50.7	26.9%	14.9%
Liberia	220	278	3.1	3.9	1.7	1.5	32.7	51.7	5.8%	3.8%
Malawi	337	257	4.0	3.0	2.6	1.8	33.2	42.0	3.3%	1.2%
Namibia	2,306	633	2.9	0.8	14.7	3.4	39.3	50.5	6.5%	3.3%
Niger	401	533	5.1	6.8	2.8	3.0	34.3	45.4	15.4%	8.9%
Nigeria	460	460	0.9	0.9	2.9	2.8	38.8	46.3	2.1%	1.1%
Senegal	819	647	4.6	3.6	5.6	3.2	35.1	48.4	14.8%	8.7%
Sierra Leone	334	353	2.3	2.4	2.8	1.8	31.3	50.4	21.2%	8.1%
Tanzania	805	773	3.8	3.7	5.0	3.9	42.4	53.2	22.5%	7.6%
Uganda	321	296	2.3	2.1	2.1	1.5	39.5	50.0	47.1%	20.9%
Zambia	1,639	465	5.1	1.5	10.7	2.3	39.2	52.1	10.1%	5.2%
**Average**	**677**	**403**	**3.2**	**2.4**	**4.7**	**2.2**	**36.1**	**49.0**	**18.6%**	**10.3%**

Notes: All the earnings are in PPP ($,2011). Teachers include survey respondents who self-identified as a primary or secondary school teacher and a wage worker. Other wage workers include respondents who self-identified as a wage worker in other occupations; university faculty, special education teachers and other teachers were excluded from the group of other wage workers. Both the teacher sample and other wage worker sample only include workers with secondary or post-secondary education and those who reported both earnings and hours worked weekly. The top and bottom 1% earnings are trimmed. The survey in The Gambia did not report weekly working hours. *Source:* Surveys listed in Table 1.

The median earnings of teachers identified in our study surveys are about $677 per month (measured at the 2011 purchasing power parity (PPP) exchange rate) (Table 4, column 1). There is, however, very large cross-country variation in these monthly earnings. Teachers make $100 per month in the Democratic Republic of Congo, $220 in Liberia, $401 in Niger, $656 in Ghana, $805 in Tanzania, $1,639 in Zambia, and $2,306 in Namibia.

**Fig. 2:**
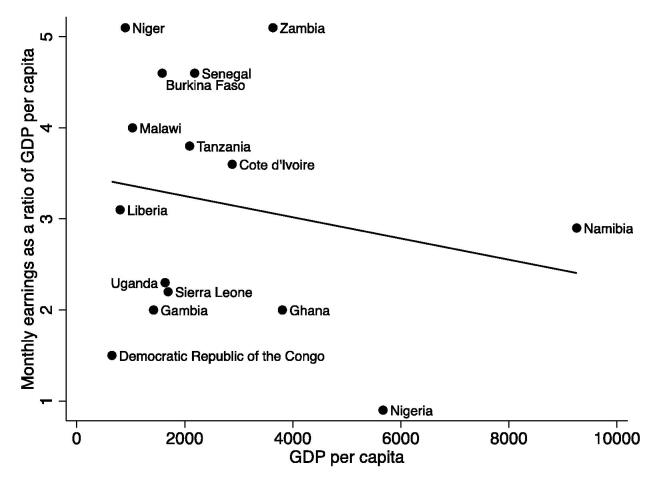
Teacher monthly earnings as a ratio of GDP per capita. Note: The solid line is the line of best fit. Sources: See Table 1 for survey data sources and year. GDP per capita data are from World Development Indicators (World Bank, 2019b).

One common way to put these numbers in context is to express teacher earnings in relation to GDP per capita. Column 3 of Table 4 reports that, on average, teachers in the sample earn 300 percent of GDP per capita, again with large variation across countries. Teachers in Niger and Zambia earn more than 500 percent of GDP per capita and, at the other extreme, teachers in Nigeria earn only 90 percent of GDP per capita. In our sample, teacher earnings and GDP per capita are highly correlated. In the OECD, teachers earn about 130 percent of GDP per capita (OECD, 2011). Plotting these numbers by countries’ GDP per capita (Fig. 2), we find suggestive evidence of a negative correlation between teacher monthly earnings as a ratio of GDP per capita and a country’s income level. In other words, teachers’ earnings rise as national income per capita rises, but at a slower rate than national incomes per capita.^25^

Teachers have higher monthly earnings than other wage workers with similar educational background in ten countries in our sample (Table 4, columns 1–4). Teachers in all countries in our sample report working significantly fewer hours per week, about 36 h among teachers compared to 49 h among other workers (Table 4, columns 7–8). After scaling monthly earnings by hours worked, the results show that teachers are paid more on an hourly basis than other workers in every country except the Democratic Republic of Congo and Niger. Furthermore, teachers are more likely to have a second job in almost every country, which suggests that teachers may want (or need) to work more to gain higher overall income (Table 4, last two columns).^26^ However, we do not observe an association between reported hours worked by teachers and the propensity to take a second job (corr = 0.09, p-value = 0.77, N = 14).

Who are these other wage workers? To better understand the relationship between teacher earnings and that of other secondary and post-secondary graduates, we summarize the broad occupations of these other workers and their earnings, separated into those with secondary and those with post-secondary education. We classify occupations using the International Labor Organization codes from the household surveys (ILO, 2012). While there is variation, across our study countries we see that among wage workers with secondary education, only one occupation – managers – earns substantially more than teachers (Table 5A). (Professionals and technicians make slightly more than teachers.) Among wage workers with post-secondary education, there are just four occupations that earn more than teachers: managers, professionals, technicians, and skilled agricultural workers (Table 5B).^27^

**Table 5A:** Monthly median earnings for workers with secondary education by occupation group.

		Other wage workers with secondary school education								
Country	Teachers	Managers	Professionals	Technicians	Clerks	Services and sales workers	Skilled agricultural workers	Craft workers	Machine operators	Elementary occupations
	(1)	(2)	(3)	(4)	(5)	(6)	(7)	(8)	(9)	(10)
Burkina Faso	603	*603*	*777*	464	441	200	*172*	181	–	–
Côte d'Ivoire	852	*574*	832	675	584	464	216	300	*777*	262
DR Congo	96	100	91	91	102	109	91	154	182	112
The Gambia	254	400	265	273	235	204	255	204	204	153
Ghana	278	556	389	367	333	222	194	339	333	233
Liberia	207	431	237	279	306	222	278	296	301	176
Malawi	258	544	243	–	254	147	176	235	235	180
Namibia	1,447	1,537	1,265	1,009	723	362	330	542	814	362
Niger	338	*889*	*267*	*886*	544	336	273	367	–	–
Nigeria	230	*402*	259	253	368	172	*92*	460	459	239
Senegal	688	1,034	690	739	647	345	334	371	323	194
Sierra Leone	301	*222*	356	319	323	289	–	278	278	222
Tanzania	724	724	–	757	600	563	*402*	644	584	394
Uganda	188	*494*	*395*	491	*79*	166	*197*	232	286	158
Zambia	1,610	1,073	1,646	1,073	716	304	206	465	465	233
**Average**	**538**	**639**	**551**	**548**	**417**	**274**	**230**	**338**	**403**	**224**

Notes: All the earnings are in PPP ($,2011). Teachers include survey respondents who self-identified as a primary or secondary school teacher and a wage worker. Other wage workers include respondents who self-identified as a wage worker in other corresponding occupation groups; university faculty, special education teachers and other teachers were excluded from the group of other wage workers. ISCO-08 group definitions are used to classify occupation groups (ILO, 2012). Elementary occupations involve the performance of simple and routine tasks which may require the use of hand-held tools and considerable physical effort. Earnings in italics are estimated based on fewer than 10 observations. - indicates no observations in that category. Sample only includes workers with secondary education. The top and bottom 1% earnings are trimmed. *Source:* Surveys listed in Table 1.

**Table 5B:** Monthly median earnings for workers with post-secondary education by occupation group.

		Other workers with post-secondary school education								
	Teachers	Managers	Professionals	Technicians	Clerks	Services and sales workers	Skilled agricultural workers	Craft workers	Machine operators	Elementary occupations
	(1)	(2)	(3)	(4)	(5)	(6)	(7)	(8)	(9)	(10)
Burkina Faso	756	*1,152*	1,508	928	673	255	*139*	*255*	–	–
Côte d'Ivoire	889	*1,229*	1,135	*638*	977	794	*1,695*	*647*	*1,038*	*349*
DR Congo	109	136	145	130	182	127	167	170	245	200
The Gambia	286	581	362	408	328	265	–	281	*235*	*174*
Ghana	778	1,111	889	778	686	378	*249*	417	667	428
Liberia	231	648	498	608	*555*	393	*648*	*278*	*200*	*197*
Malawi	330	1,652	751	–	713	520	*419*	419	*384*	*118*
Namibia	2,532	3,978	2,893	2,210	1,266	1,422	*4,069*	1,686	1,655	*814*
Niger	374	*2,133*	1,444	1,304	800	551	*946*	*944*	–	–
Nigeria	483	796	828	598	525	*443*	*667*	828	*345*	374
Senegal	862	1,724	1,293	948	862	733	*1,293*	*754*	*216*	*1,185*
Sierra Leone	350	834	489	501	417	381	*1,669*	445	*620*	*195*
Tanzania	831	1,288	1,288	905	966	857	–	825	*805*	–
Uganda	336	541	494	494	395	355	*197*	415	–	276
Zambia	1,717	1,725	1,789	1,646	1,302	*537*	*1,413*	1,673	1,073	1,285
**Average**	**724**	**1,302**	**1,054**	**864**	**710**	**534**	**1,044**	**669**	**624**	**466**

Notes: All the earnings are in PPP ($,2011). Teachers include survey respondents who self-identified as a primary or secondary school teacher and a wage worker. Other wage workers include respondents who self-identified as a wage worker in other corresponding occupation groups; university faculty, special education teachers and other teachers were excluded from the group of other wage workers. ISCO-08 group definitions are used to classify occupation groups (ILO, 2012). Elementary occupations involve the performance of simple and routine tasks which may require the use of hand-held tools and considerable physical effort. Earnings in italics are estimated based on fewer than 10 observations. - indicates no observations in that category. Sample only includes workers with post-secondary education. The top and bottom 1% earnings are trimmed. *Source:* Surveys listed in Table 1.

A related question to the level of earnings is whether earnings are more or less explained by observable characteristics for teachers as compared to other occupations. Since teacher salaries are often set centrally according to qualifications and experience, one might expect that they should be more readily explained by these attributes. To explore this, we regress monthly earnings on gender, age (and its square), education, and urban status separately for teachers and for other wage workers and compare the R-squared (R^2^), a measure of how much variation is captured by these observed characteristics (results are reported in Appendix Table A18). Perhaps surprisingly given the expected link between teacher salary and characteristics, we find that in our study countries teacher earnings are slightly less explained by observed characteristics (R^2^ of 0.168) than those of other wage workers (R^2^ of 0.250). Even in Ghana, the country in our sample with the highest R^2^ for teachers, less than half of teacher earnings are explained by observed characteristics. These statistics are not consistent with the idea that teacher pay is much more likely to be determined by age and education than other wage occupations in these countries.

If we divide teachers by contract type, we find that, on average, the median monthly earnings of fixed term contract teachers are just 70 percent of the earnings of permanent teachers (Table 6). The ratio ranges from just 33 percent in Côte d'Ivoire to 93 percent in the Democratic Republic of Congo. The range is similar for hourly earnings, suggesting that teachers on permanent and fixed term contracts report similar working hours (Table 7).

**Table 6:** Comparison of teacher monthly earnings by contract type.

	Teachers on a permanent contract					Teachers on a fixed term/temporary contract					
	Median	Standard deviation	Inter-quartile	Inter-quartile/mean	N	Median	Standard deviation	Inter-quartile	Inter-quartile/mean	N	Ratio of medians
	(1)	(2)	(3)	(4)	(5)	(6)	(7)	(8)	(9)	(10)	(11)
Burkina Faso	650	733	557	0.88	251	232	464	668	1.4	24	0.36
Côte d'Ivoire	1,059	627	508	0.46	49	350	401	640	1.3	34	0.33
DR Congo	100	60	18	0.18	544	92	56	41	0.4	373	0.93
The Gambia	N.A.					N.A.					
Ghana	N.A.					N.A.					
Liberia	N.A.					N.A.					
Malawi	N.A.					N.A.					
Namibia	2,351	894	1,085	0.48	291	1,537	1029	1,772	1.0	58	0.65
Niger	542	1902	556	0.57	78	334	179	57	0.2	36	0.62
Nigeria	N.A.					N.A.					
Senegal	832	633	388	0.43	224	690	1,301	388	0.4	54	0.83
Sierra Leone	334	6,007	233	0.20	176	278	7,620	342	0.2	21	0.83
Tanzania	805	370	407	0.47	363	713	679	459	0.5	20	0.89
Uganda	345	147	94	0.25	95	252	243	222	0.7	66	0.73
Zambia	1,691	579	465	0.27	411	1,431	820	1,360	1.1	59	0.85
**Average**	**871**	**1,195**	**431**	**0.42**	**248**	**591**	**1,279**	**595**	**0.7**	**75**	**0.70**

Notes: Earnings are in PPP ($,2011). Ratio of medians is the ratio of the median earnings for teachers on a fixed term or temporary contract to that for teachers on a permanent contract. Teachers include survey respondents who self-identified as a primary or secondary school teacher and a wage worker. All teachers have secondary or secondary or post-secondary education. The top and bottom 1% earnings are trimmed. Surveys in The Gambia, Ghana, Liberia, Malawi, and Nigeria did not report whether a worker was on a permanent or fixed term/temporary contract. *Source:* Surveys listed in Table 1.

**Table 7:** Comparison of teacher hourly earnings by contract type.

	Teachers on a permanent contract					Teachers on a fixed term/temporary contract					
	Median	Standard deviation	Inter-quartile	Inter-quartile/mean	N	Median	Standard deviation	Inter-quartile	Inter-quartile/mean	N	Ratio of medians
	(1)	(2)	(3)	(4)	(5)	(6)	(7)	(8)	(9)	(10)	(11)
Burkina Faso	4.0	4.9	3.9	0.94	247	1.5	3.8	4.5	1.3	24	0.38
Côte d'Ivoire	7.6	9.9	5.9	0.53	48	3.8	4.2	3.2	0.7	32	0.50
DR Congo	0.7	0.6	0.4	0.44	537	0.7	0.5	0.4	0.6	367	1.00
The Gambia	N.A.					N.A.					
Ghana	N.A.					N.A.					
Liberia	N.A.					N.A.					
Malawi	N.A.					N.A.					
Namibia	14.8	6.4	7.6	0.51	291	9.6	6.4	10.8	1.0	58	0.65
Niger	4.2	14.1	4.6	0.59	78	2.4	2.1	0.7	0.2	36	0.57
Nigeria	N.A.					N.A.					
Senegal	5.9	5.8	5.2	0.67	224	4.5	6.9	3.5	0.6	54	0.76
Sierra Leone	2.6	60.2	2.2	0.13	127	4.0	87.2	4.3	0.1	11	1.51
Tanzania	5.0	2.3	2.7	0.51	363	4.0	2.8	2.7	0.6	20	0.80
Uganda	2.3	3.2	1.3	0.40	92	1.5	1.9	1.6	0.7	62	0.64
Zambia	11.2	4.4	4.5	0.39	411	8.3	5.0	8.5	1.1	59	0.75
**Average**	**5.8**	**11.2**	**3.8**	**0.51**	**242**	**4.0**	**12.1**	**4.0**	**0.7**	**72**	**0.76**

Notes: Earnings are in PPP ($,2011). Ratio of medians is the ratio of the median earnings for teachers on a fixed term or temporary contract to that for teachers on a permanent contract. Teachers include survey respondents who self-identified as a primary or secondary school teacher and a wage worker. All teachers have secondary or secondary or post-secondary education. The top and bottom 1% earnings are trimmed. Surveys in The Gambia, Ghana, Liberia, Malawi, and Nigeria did not report whether a worker was on a permanent or fixed term/temporary contract. *Source:* Surveys listed in Table 1.

### 3.4 How are teachers paid relative to other professionals, adjusting for controls?

#### 3.4.1 Monthly earnings

Table 8 reports the monthly earnings differentials estimated by multivariate regression analyses which control for education, age (and its square), gender, and rural/urban location. We first estimate the model for all teachers using OLS and median regression (Columns 1 and 2 respectively). We then replace the teacher dummy with primary-teacher and secondary-teacher dummies in the OLS regression (Columns 3 and 4). Next, we keep only teachers in public schools and remove all other teachers from the sample (Column 5). Last, we estimate the same model but limit the sample to public sector employees to compare how public school teachers are paid compared to other public sector employees (Column 6). As discussed above, for this multivariate analysis we restrict the sample to wage workers with secondary education or more.

**Table 8:** Monthly earnings differentials between teachers and other wage workers.

	All teachers		Primary teachers	Secondary teachers	Public school teachers	Public school teachers relative to other public sector employees
	OLS	Median	OLS			
	(1)	(2)	(3)	(4)	(5)	(6)
Burkina Faso	0.433***	0.616***	0.447***	0.358	0.495***	0.344***
	(0.104)	(0.090)	(0.111)	(0.238)	(0.113)	(0.132)
Côte d'Ivoire	0.287**	0.437***	0.342**	0.222	0.648***	0.180
	(0.117)	(0.114)	(0.154)	(0.166)	(0.135)	(0.143)
DR Congo	−0.121***	−0.099***	−0.113***	−0.159**	−0.135***	−0.084**
	(0.038)	(0.029)	(0.040)	(0.079)	(0.040)	(0.040)
The Gambia	−0.184***	−0.182***	−0.343***	0.049	−0.143**	−0.142*
	(0.056)	(0.050)	(0.069)	(0.081)	(0.069)	(0.077)
Ghana	0.051	−0.004	0.022	0.093	0.128***	−0.076
	(0.045)	(0.045)	(0.055)	(0.063)	(0.050)	(0.056)
Liberia	−0.475***	−0.337***	−0.433***	−0.642***	−0.398***	−0.405***
	(0.084)	(0.083)	(0.092)	(0.172)	(0.098)	(0.105)
Malawi	0.018	0.143**	N.A.		0.075	−0.008
	(0.058)	(0.059)			(0.063)	(0.072)
Namibia	0.381***	0.352***	0.390***	0.365***	0.409***	0.238***
	(0.058)	(0.072)	(0.068)	(0.087)	(0.059)	(0.055)
Niger	−0.156	−0.162*	−0.042	−0.510*	−0.026	−0.275
	(0.140)	(0.092)	(0.157)	(0.262)	(0.154)	(0.200)
Nigeria	−0.250***	−0.276***	−0.297***	−0.166	−0.043	−0.172**
	(0.071)	(0.072)	(0.082)	(0.109)	(0.078)	(0.071)
Senegal	0.247***	0.202***	0.330***	0.132	0.300***	0.155***
	(0.055)	(0.059)	(0.070)	(0.081)	(0.057)	(0.055)
Sierra Leone	−0.586***	−0.286***	−0.660***	−0.480***	−0.541***	−0.517***
	(0.132)	(0.106)	(0.158)	(0.182)	(0.145)	(0.165)
Tanzania	−0.143***	−0.088**	−0.162***	−0.099	−0.139***	−0.245***
	(0.040)	(0.040)	(0.046)	(0.065)	(0.042)	(0.041)
Uganda	−0.188**	−0.301***	−0.240***	0.075	−0.169*	−0.413***
	(0.078)	(0.087)	(0.081)	(0.148)	(0.092)	(0.102)
Zambia	0.522***	0.391***	0.490***	0.608***	0.610***	0.218***
	(0.045)	(0.058)	(0.051)	(0.076)	(0.048)	(0.042)
**Average**	**−0.011**	**0.027**	**−0.019**	**−0.011**	**0.071**	**−0.080**

Notes: Each column reports the coefficient in the regressions of ln (monthly earnings) on (a dummy for) a type of teachers with controls. The comparison group are other wage workers in columns 1–5 and other public sector employees in column 6. Controls include gender, age, age-squared, urbanicity and (a dummy for) post-secondary education. Sample is restricted to workers with secondary education or more. The survey in Malawi did not differentiate the levels of teachers. Standard errors in parentheses. *** p < 0.01, ** p < 0.05, * p < 0.1. *Source:* Surveys listed in Table 1.

In five of the 15 countries in the sample (Burkina Faso, Côte d’Ivoire, Namibia, Senegal and Zambia), teachers have a statistically significant monthly earnings premium that averages 37.4 percent. In three of the countries (Ghana, Malawi, and Niger), teachers earn similar amounts to comparable wage workers. In the remaining seven countries, teachers exhibit a statistically significant deficit in earnings relative to comparable wage workers that averages 28 percent. This basic set of results is robust to whether we alternatively use OLS on a trimmed sample (removing the top and bottom 1% of earnings) or even median regression on the trimmed sample, although the point estimates vary for some countries (Appendix Table A4, Table A5, Table A6, Table A7, Table A8).^28^

Given that most teachers are in primary schools, and that most are in public schools, it is unsurprising that the results are similar when we focus on those subsets of the samples. The results suggest that, in general, when public sector teachers have an earnings premium relative to comparable workers, they also have a premium relative to other public sector employees—although this premium is smaller. In these five countries, therefore, this indicates a “hierarchy” of premia (with teachers having the higher premium). In countries where public sector teachers have an earnings deficit relative to comparable workers, they also tend to face a deficit relative to other public sector employees (with these magnitudes often being similar).

The results for secondary school teachers are different: in many more cases these teachers are paid similarly to comparable workers. There are four countries (out of 14 for which we can estimate this) where there is a statistically significant earnings deficit for teachers (which averages 45 percent); two countries where there is a premium for teachers; and in the remaining eight countries there is no statistically significant difference.

#### 3.4.2 Hourly earnings

With respect to hourly earnings, the patterns are more systematic. Teachers have a statistically significant hourly-earnings premium in seven of the 14 African countries. Hourly-earnings deficits are rare, but nevertheless evident in Nigeria (Table 9). Averaging across all values (positive and negative, statistically significant and non-significant), the hourly wage premium for teachers in these African countries is 26 percent. Recall that in monthly earnings terms these averages were close to zero (which masked substantial heterogeneity). The overall pattern is once again similar when using trimmed or median regression.

**Table 9:** Hourly earnings differentials between teachers and other wage workers.

	All teachers		Primary teachers	Secondary teachers	Public school teachers	Public school teachers relative to other public sector employees
	OLS	Median	OLS			
	(1)	(2)	(3)	(4)	(5)	(6)
Burkina Faso	0.631***	0.763***	0.652***	0.517**	0.696***	0.491***
	(0.107)	(0.093)	(0.114)	(0.245)	(0.117)	(0.134)
Côte d'Ivoire	0.734***	0.794***	0.658***	0.826***	1.023***	0.540***
	(0.129)	(0.131)	(0.168)	(0.184)	(0.149)	(0.160)
DR Congo	0.043	0.107***	0.039	0.063	0.017	0.018
	(0.040)	(0.040)	(0.043)	(0.085)	(0.043)	(0.044)
The Gambia	N.A.					

Ghana	0.324***	0.281***	0.294***	0.364***	0.435***	0.234***
	(0.051)	(0.054)	(0.063)	(0.072)	(0.057)	(0.060)
Liberia	−0.059	0.023	−0.034	−0.158	0.041	0.011
	(0.091)	(0.081)	(0.099)	(0.186)	(0.106)	(0.120)
Malawi	0.169**	0.198***	N.A.		0.225***	0.124
	(0.073)	(0.073)			(0.080)	(0.092)
Namibia	0.563***	0.522***	0.597***	0.502***	0.581***	0.383***
	(0.062)	(0.077)	(0.073)	(0.093)	(0.064)	(0.058)
Niger	0.096	0.061	0.158	−0.098	0.199	−0.089
	(0.145)	(0.100)	(0.163)	(0.271)	(0.159)	(0.205)
Nigeria	−0.146*	−0.180**	−0.206**	−0.020	0.061	−0.058
	(0.086)	(0.079)	(0.099)	(0.131)	(0.095)	(0.088)
Senegal	0.564***	0.571***	0.587***	0.530***	0.620***	0.431***
	(0.059)	(0.063)	(0.075)	(0.087)	(0.061)	(0.058)
Sierra Leone	0.047	−0.035	0.094	−0.037	−0.020	−0.016
	(0.167)	(0.131)	(0.195)	(0.246)	(0.188)	(0.223)
Tanzania	0.003	0.022	−0.010	0.035	0.020	−0.138***
	(0.044)	(0.050)	(0.050)	(0.072)	(0.046)	(0.043)
Uganda	−0.027	−0.142	−0.085	0.260	0.017	−0.138
	(0.095)	(0.101)	(0.100)	(0.180)	(0.111)	(0.144)
Zambia	0.697***	0.524***	0.659***	0.797***	0.785***	0.349***
	(0.050)	(0.063)	(0.056)	(0.084)	(0.053)	(0.045)
**Average**	**0.260**	**0.251**	**0.262**	**0.275**	**0.336**	**0.153**

Notes: Each column reports the coefficient in the regressions of ln (monthly earnings) on (a dummy for) a type of teachers with controls. The comparison group are other wage workers in columns 1–5 and other public sector employees in column 6. Controls include gender, age, age-squared, urbanicity and (a dummy for) post-secondary education. Sample is restricted to workers with secondary education or more. The survey in Malawi did not differentiate the levels of teachers and the survey in The Gambia did not report weekly earnings. Standard errors in parentheses. *** p < 0.01, ** p < 0.05, * p < 0.1. *Source:* Surveys listed in Table 1.

The results are also similar when restricting the analysis to primary school teachers or to public school teachers. Public sector teachers tend to have either no premium or a positive hourly earnings premium when compared to other public sector employees (13 of the 14 countries for which this could be estimated). Only in Tanzania do they have a deficit relative to comparable civil servant employees. Last, the hourly earnings premia are typically also found for secondary school teachers (i.e., these are now more in line with teachers overall/primary school teachers).

Across contract types, earnings premia are higher (or less negative) for teachers with permanent contracts than for teachers with fixed-term contracts. For both monthly and hourly wages, there are more statistically significant premia for permanent contract teachers than for fixed-term contract teachers (Table 10).

**Table 10:** Earnings differentials between teachers and other wage workers by contract type.

	Monthly			Hourly	
	Permanent	Fixed term/temporary		Permanent	Fixed term/temporary
	(1)	(2)		(3)	(4)
Burkina Faso	0.474***	0.042		0.656***	0.416
	(0.107)	(0.305)		(0.110)	(0.311)
Côte d'Ivoire	0.625***	−0.113		1.024***	0.357*
	(0.143)	(0.168)		(0.157)	(0.185)
DR Congo	−0.061	−0.152***		0.089*	0.020
	(0.045)	(0.054)		(0.048)	(0.058)
The Gambia	N.A.				
Ghana	N.A.				
Liberia	N.A.				
Malawi	N.A.				
Namibia	0.393***	0.279**		0.566***	0.478***
	(0.062)	(0.123)		(0.066)	(0.132)
Niger	−0.098	−0.252		0.157	−0.039
	(0.165)	(0.231)		(0.169)	(0.240)
Nigeria	N.A.				
Senegal	0.234***	0.178		0.568***	0.417***
	(0.059)	(0.114)		(0.063)	(0.122)
Sierra Leone	−0.558***	−0.709**		0.016	0.493
	(0.137)	(0.322)		(0.171)	(0.461)
Tanzania	−0.141***	−0.258*		0.011	−0.210
	(0.041)	(0.152)		(0.045)	(0.168)
Uganda	−0.192**	−0.180*		0.020	−0.100
	(0.097)	(0.104)		(0.118)	(0.123)
Zambia	0.589***	0.036		0.762***	0.179
	(0.048)	(0.109)		(0.053)	(0.122)
**Average**	**0.127**	**−0.113**		**0.387**	**0.201**

Notes: Each column reports the coefficient in the regressions of ln (monthly/hourly earnings) on (a dummy for) a type of teachers with controls. The comparison group are other wage workers. Controls include gender, age, age-squared, urbanicity and (a dummy for) post-secondary education. Sample is restricted to workers with secondary education or more. Surveys in The Gambia, Ghana, Liberia, Malawi, and Nigeria did not report whether a worker was on a permanent or fixed term/temporary contract. Standard errors in parentheses. *** p < 0.01, ** p < 0.05, * p < 0.1. *Source:* Surveys listed in Table 1.

#### 3.4.3 Robustness to inclusion of non-pecuniary benefits

In our data we find that teachers are significantly more likely to report receiving benefits than other workers: while teachers and other workers are comparably likely to report medical benefits, teachers are much more likely to report receiving paid leave or a pension (Table A19).^29^ On average, only teachers on fixed term (as opposed to permanent) contracts have similar (or slightly worse) benefits to other workers: for example, 50 percent of teachers on fixed term contracts report access to paid leave versus 50 percent of other wage workers (Table A19, Table A20). Teachers on fixed term contracts are slightly less likely to receive medical benefits or a pension than other workers.^30^ As a result of these benefits, our earnings differential estimates likely represent an underestimate of the teacher premium. As a summary indicator, teachers are more likely than other workers to report at least one of these supplementary benefits (paid leave, medical benefits, or a pension) in 11 of the 13 countries for which surveys report benefits (Table A21). In the two exceptions, the Democratic Republic of the Congo and Liberia, teachers have almost an identical likelihood of reporting benefits relative to other workers.

In virtually every case in our sample, a simple regression of whether or not a teacher or other worker reports receiving at least one benefit on their earnings (together with controls for age, gender, urban/rural residence, and post-secondary education) suggests that non-pecuniary compensation and pay are complements for both teachers and other workers: in other words, those who are more likely to receive non-pecuniary compensation are also more likely to receive higher pay (Table A21). While there is variation across countries, in most cases the complementarity is stronger among other workers than among teachers, which suggests that even at lower levels of pay, teachers are likely to receive non-pecuniary benefits.

How are these benefits likely to affect our estimates of teacher compensation? Once we adjust for hours reported (discussed in section 3.4.2 above), we find that only in Nigeria do teachers report a significant earnings deficit relative to other workers with similar qualifications (Table 9). But in Nigeria, teachers are almost 16 percent more likely to report receiving at least one benefit than other workers (Table A21, column 4). Specifically, they are much more likely to report receiving a pension (Table A19), although they are less likely to report medical benefits. On net, however, it is plausible that this one negative earnings differential is reduced or eliminated by non-pecuniary benefits. The country with the next lowest point estimate in terms of relative hourly earnings is Liberia, where the difference is not statistically significant (Table 9). In that case, teachers and other workers report similar rates of benefits (Table A19; Table A21), such that teachers likely remain comparably paid to other workers once non-pecuniary benefits are incorporated.

Thus, the evidence on non-pecuniary benefits, together with the analysis of hours worked, suggest that it is likely that in no country in our sample are teachers paid worse than other workers on an hourly basis.

### 3.5 Exploring the heterogeneity in results

While we do not see consistent evidence in our sample that teachers have a negative earnings differential relative to other workers once one adjusts for hours and non-pecuniary benefits, we do observe a high degree of variation in the earnings differential—from −59 percent to + 52 percent in monthly earnings and from −15 percent to + 73 percent in hourly earnings. In this section, we explore factors of the economy and of the education system that could theoretically explain some of this differential (Table A23, Table A24). The first takeaway is that the teacher earnings differential is not the only way in which these economies and education systems vary. We observe enormous variation in every aspect we examine. For example, the ratio of GDP per capita in the richest economy in our sample (Namibia) to that of the poorest (the Democratic Republic of the Congo) is more than 14 (Table A23). The ratio of the highest versus lower share of wage workers in the labor force (an indicator of the formality of the economy) is 4.5, and female labor force participation is double in our highest country (Côte d’Ivoire) versus our lowest (Senegal). The education systems likewise vary enormously (Table A24), with a 25-year span across which some form of free primary education was introduced, massive variation in teacher unionization (from 90 percent in Tanzania to 36 percent in Côte d’Ivoire), and private school enrollments ranging from 2.5 percent (Malawi) to 41 percent (Liberia).

Given this massive variation in economies and education systems, it is perhaps unsurprising that teacher pay premia should vary as well. However, we find little evidence that observed characteristics of the economy or of the education system explain teacher earnings premia. We present correlations between each characteristic that we observe and the monthly or hourly earnings premium. Across all the characteristics we measure, we observe just one statistically significant correlation. This general lack of statistical significance sets the tone for our subsequent discussion of the patterns we observe in that the correlations as purely suggestive.

What might explain heterogeneity in teacher earnings premia? Wealthier economies may have better developed public sectors and so may pay their teachers more. We observe a weak positive correlation between GDP per capita and teacher wage premia (both monthly and hourly) (Table A23). In Section 3.3, we identified that teacher earnings rise with average incomes; here we add that premia may also rise with GDP per capita. We also see a weak positive correlation with the share of wage workers in the labor force, a measure of the relative size of the formal sector. Female labor force participation is not correlated with teacher earnings premia.

In terms of the education sector, a more educated teacher workforce could potentially command higher returns. We observe a moderate positive (and statistically significant at the 5 percent level) correlation between years of schooling of teachers relative to those for other workers and earnings premia (both monthly and hourly) (Table A24). We also observe a weak positive association between the likelihood of teachers having at least one non-pecuniary benefit relative to other workers and the wage premia, suggesting that premia may be complements to other benefits. We have data on teacher unionization from only six countries, but that association is weakly positive for monthly wages and negative for hourly wages, suggesting perhaps that teacher unions are more effective at negotiating over hours than wages.

Does competition from the private sector lead to higher premia for teachers? We observe very weak correlations with the share of the private sector in education. Likewise, we observe nearly zero correlation with the teacher share of the labor force, which might be expected to drive down earnings. The same is true for the relative growth rate of teachers as a fraction of the labor force. Finally, we see very weak correlations with the year of free primary education (FPE), which might indicate a more experienced body of teachers (i.e., because of a large group hired at the time of FPE).

Ultimately, this exercise demonstrates that teacher earnings premia cannot be explained by some of the most obvious candidates within either the economy or the education system. As these are simple correlations, the lack of significance suggests that even other, unobserved characteristics that are highly correlated with the above are unlikely to explain teacher earnings premia. Rather, these premia are likely determined by a more complex combination of factors—including political processes—idiosyncratic to each country’s history.

## 4 Conclusion

Teacher pay makes up the vast majority of education budgets in many African countries (Ugandan Ministry of Education and Sport and UNESCO IIEP Pôle de Dakar, 2014, World Bank, 2018). In this paper, we present new evidence on teacher earnings across a set of 15 Sub-Saharan African countries. We document key descriptive statistics about the level of teacher earnings and compare this level to earnings for other workers of comparable education and experience in the same economies. Our results may explain part of the contention between those who argue that teachers are paid too little and those who argue that teachers are paid too much (Das, 2017). In several countries in our sample, teachers earn less than comparable workers in terms of their monthly earnings, but they earn more than others per hour. We document that teachers receive a wide range of benefits that other workers tend not to receive, so earnings estimates for teachers should be seen as underestimates. Incorporation of those benefits into earnings would likely mean that in only one or none of the countries in our sample do teachers earn less on an hourly basis than other workers with similar years of education. In several countries, the earnings gap between teachers on fixed term and permanent contracts is large. Teachers are much more likely to take a second job than other workers.

There is a high degree of heterogeneity in teacher earnings premia. These results highlight the danger of adopting a single narrative about compensation for teachers or perhaps even other public sector workers. Extrapolating from a single case—whether it is Côte d’Ivoire or Nigeria—is unlikely to be instructive when teacher pay differentials and the structure of teacher pay vary so much from country to country, even on the same region or continent. The variation in earnings across teachers—even restricting only to teachers on permanent contracts—differs greatly, from low variation in Sierra Leone to high variation in Burkina Faso. The most obvious candidates to explain some of this variation are not statistically significantly correlated with teacher earnings premia. Ultimately, there is no clear, single narrative on teacher compensation that explains the wide variation in the structure of teacher labor markets.

Much of this paper focuses on national differences, but there are important subnational differences beyond the teacher contract differences we have discussed. Many countries provide earnings allowances to teachers who work in rural schools (Evans & Mendez Acosta, 2021a). Evidence from the Gambia and Zambia suggests that these programs can be effective at reducing teacher shortages (Chelwa et al., 2019, Pugatch and Schroeder, 2014). However, the amount affects the impact: research from Malawi suggests that allowances were too small to draw teachers to rural areas (Mwenda & Mgomezulu, 2018). Furthermore, while our paper focuses on teacher pay levels, teachers face other challenges in low- and middle-income countries, including delays in payment (Evans & Yuan, 2018).

Teacher pay clearly matters: it occupies most of the education budget, it has implications for who selects into the teaching profession, who stays in that profession, and who might be willing to work in difficult-to-staff schools. However, teacher earnings relative to other professionals varies across countries, across types of contracts, and across who one uses as a comparison. Although some research suggests an association between teacher earnings and student learning outcomes, enough well causally identified research casts doubt on that association to suggest that the link is at the very least not simple. Higher wages are a blunt instrument for inviting better candidates to the teaching profession, but other approaches—e.g., seeking to select better candidates via observable characteristics—have limitations in that most observed teacher characteristics explain relatively little of teacher value added in test scores (Cruz-Aguayo et al., 2017, Hanushek and Rivkin, 2006). Deepening our understanding of what drives teacher pay and—in turn—what teacher pay affects will be crucial as countries across Africa expand their teaching workforce in the coming years.

## Declaration of Competing Interest

The authors declare that they have no known competing financial interests or personal relationships that could have appeared to influence the work reported in this paper.

## Figures and Tables

**Table A1:** Data processing notes.

Country	Source	Year	Occupation code type	Weekly hours reported	Reported income from main job	Reported secondary job
	(1)	(2)	(3)	(4)	(5)	(6)
Burkina Faso	Continuous Multisectoral Survey	2014	Survey-specific, 4 digits	From daily hours and working days in a week	Earnings + period	Yes
Côte d'Ivoire	National Employment Survey	2013	Survey-specific, 3 digits	From daily hours and working days in a week	Earnings + period	Yes
DR Congo	National Household Survey	2012	Survey-specific, 3 digits	Reported	Monthly earnings	Yes
The Gambia	Integrated Household Survey	2010	ISCO codes (4 digits)	Working hours not reported	Earnings + period	No
Ghana	Ghana Living Standards Survey 6	2012–2013	ISCO codes (4 digits)	Reported	Earnings + period	Yes
Liberia	Household Income and Expenditure Survey	2014–2015	ISCO 08 code (4 digits)	Reported	Earnings + period	Yes
Malawi	Integrated Household Panel Survey	2010	Survey-specific major groups, 2 digit group	Reported	Earnings + period	Yes
Namibia	Labor Force Survey	2013	ISCO codes (4 digits)	Reported	Earnings + period	Yes
Niger	National Survey on Household Living Conditions and Agriculture	2014	Survey-specific, 4-digit	Reported	Earnings + period	Yes
Nigeria	General Household Survey	2015	ISCO codes (4 digits)	Reported	Earnings + period	Yes
Senegal	Poverty Monitoring Survey	2010	Survey-specific, 3 digits	Reported (but both primary and secondary jobs)	Monthly	Yes
Sierra Leone	Labor Force Survey	2014	ISCO codes (4 digits)	Reported	Earnings + period	Yes
Tanzania	Labor Force Survey	2014	country-specific, TASCO (4 digits)	Reported	Earnings + period	Yes
Uganda	Living Standards Measurement Survey	2013	ISCO codes, 4 digits	Reported	Earnings + period	Yes
Zambia	Labor Force Survey	2014	ISCO codes (4 digits)	From daily hours and working days in a week	Earnings + period	Yes

Notes: The surveys are publicly available in the World Bank Microdata Library. Column 4 describes how weekly working hours were calculated for each survey. Column 5 refers to how income was reported in each survey; “Earnings + period” mean that a survey reported earnings and its corresponding working period (i.e., hours, days, weeks, trimesters or year).

**Table A2:** Reports of in-kind payments.

Country	Source	Year	In-kind payment	% teachers reported in-kind payment	Share of in-kind payment in teacher earnings	% other workers reported in-kind payment	Share of other workers’in-kind payment in total earnings
		(1)	(2)	(3)	(4)	(5)	(6)
Burkina Faso	Continuous Multisectoral Survey	2014	Yes, benefits and nutrition	0.0%	–	0.1%	–
Côte d'Ivoire	National Employment Survey	2013	No	N.A.			
DR Congo	National Household Survey	2012	No	N.A.			
The Gambia	Integrated Household Survey	2010	No	N.A.			
Ghana	Ghana Living Standards Survey 6	2012–2013	Yes	1.8%	38.1%	5.8%	37.5%
Liberia	Household Income and Expenditure Survey	2014–2015	Yes, payment in any other form	8.8%	31.7%	24.6%	19.5%
Malawi	Integrated Household Panel Survey	2010	Yes, other allowance	27.4%	26.8%	20.6%	32.7%
Namibia	Labor Force Survey	2013	No, gross income before any deduction	N.A.			
Niger	National Survey on Household Living Conditions and Agriculture	2014	Yes, in-kind + nutrition payment	13.4%	18.6%	17.6%	34.0%
Nigeria	General Household Survey	2015	Yes, in-kind	10.4%	14.0%	17.1%	21.7%
Senegal	Poverty Monitoring Survey	2010	Reported total income including in-kind payment	N.A.			
Sierra Leone	Labor Force Survey	2014	Yes, in-kind + bonus	9.5%	15.3%	40.1%	27.1%
Tanzania	Labor Force Survey	2014	No	N.A.			
Uganda	Living Standards Measurement Survey	2013	Yes	40.0%	19.8%	35.1%	32.2%
Zambia	Labor Force Survey	2014	Yes	0.0%	–	1.1%	–

Notes: The surveys are publicly available in the World Bank Microdata Library. Column 2 describes whether and which in-kind payments were included in each survey. - indicates that we cannot estimate the share because so few teachers reported in-kind payments.

**Table A3:** Monthly earnings differentials between teachers and other wage workers (without in-kind payments).

	All teachers		Primary teachers	Secondary teachers	Public school teachers	Public school teachers relative to other public sector employees
	OLS	Median	OLS			
	(1)	(2)	(3)	(4)	(5)	(6)
Burkina Faso	N.A.					

Côte d'Ivoire	N.A.					

DR Congo	N.A.					

The Gambia	N.A.					

Ghana	0.059	−0.002	0.038	0.089	0.140***	−0.064
	(0.044)	(0.046)	(0.054)	(0.063)	−0.049	−0.055
Liberia	−0.445***	−0.256***	−0.404***	−0.607***	−0.347***	−0.360***
	(0.082)	(0.087)	(0.090)	(0.169)	(0.097)	(0.104)
Malawi	0.085	0.226***	N.A.			0.137**
	(0.056)	(0.055)				(0.061)
Namibia	N.A.					

Niger	−0.188	−0.151	−0.072	−0.535**	−0.054	−0.229
	(0.142)	(0.099)	(0.159)	(0.258)	(0.155)	(0.194)
Nigeria	−0.248***	−0.268***	−0.288***	−0.178*	−0.037	−0.173**
	(0.070)	(0.072)	(0.081)	(0.108)	(0.077)	(0.070)
Senegal	N.A.					

Sierra Leone	−0.443***	−0.145	−0.523***	−0.328*	−0.399***	−0.405**
	(0.127)	(0.098)	(0.152)	(0.175)	(0.139)	(0.160)
Tanzania	N.A.					

Uganda	−0.173**	−0.254***	−0.210**	0.013	−0.143	−0.408***
	(0.077)	(0.084)	(0.081)	(0.148)	(0.092)	(0.097)
Zambia	N.A.					


Notes: Each column reports the coefficient in the regressions of ln (monthly earnings) on (a dummy for) a type of teachers with controls. The comparison group are other wage workers in columns 1–5 and other public sector employees in column 6. Controls include gender, age, age-squared, urbanicity and (a dummy for) post-secondary education. The top and bottom 1% earnings are trimmed. The survey in Malawi did not differentiate the levels of teachers. The surveys in Burkina Faso, Côte d'Ivoire, DR Congo, The Gambia, Namibia, Senegal, Tanzania and Zambia did not report in-kind payments. Standard errors in parentheses. Sample is restricted to workers with secondary education or more. *** p < 0.01, ** p < 0.05, * p < 0.1. *Source:* Surveys listed in Table 1.

**Table A4:** Monthly earnings differentials between teachers and other wage workers (trimmed sample).

	All teachers		Primary teachers	Secondary teachers	Public school teachers	Public school teachers relative to other public sector employees
	OLS	Median	OLS			
	(1)	(2)	(3)	(4)	(5)	(6)
Burkina Faso	0.403***	0.629***	0.418***	0.322	0.472***	0.369***
	(0.093)	(0.088)	(0.099)	(0.211)	(0.100)	(0.121)
Côte d'Ivoire	0.382***	0.475***	0.481***	0.268*	0.700***	0.306***
	(0.102)	(0.116)	(0.134)	(0.143)	(0.118)	(0.108)
DR Congo	−0.103***	−0.090***	−0.101***	−0.109	−0.116***	−0.060
	(0.035)	(0.027)	(0.037)	(0.073)	(0.037)	(0.037)
The Gambia	−0.191***	−0.149***	−0.343***	0.033	−0.162***	−0.179***
	(0.044)	(0.050)	(0.054)	(0.063)	(0.053)	(0.056)
Ghana	−0.024	−0.023	−0.038	−0.004	0.045	−0.130***
	(0.040)	(0.045)	(0.049)	(0.057)	(0.045)	(0.047)
Liberia	−0.441***	−0.281***	−0.399***	−0.601***	−0.356***	−0.314***
	(0.069)	(0.080)	(0.075)	(0.139)	(0.080)	(0.075)
Malawi	0.022	0.140**	N.A.		0.101*	−0.036
	(0.050)	(0.057)			(0.055)	(0.057)
Namibia	0.386***	0.359***	0.391***	0.379***	0.415***	0.250***
	(0.052)	(0.071)	(0.061)	(0.078)	(0.053)	(0.048)
Niger	−0.128	−0.168*	−0.046	−0.378**	−0.007	−0.173
	(0.097)	(0.092)	(0.109)	(0.180)	(0.105)	(0.119)
Nigeria	−0.317***	−0.297***	−0.390***	−0.183**	−0.130**	−0.237***
	(0.059)	(0.072)	(0.068)	(0.090)	(0.064)	(0.059)
Senegal	0.206***	0.225***	0.284***	0.095	0.255***	0.102**
	(0.050)	(0.058)	(0.063)	(0.074)	(0.051)	(0.050)
Sierra Leone	−0.464***	−0.201*	−0.482***	−0.436***	−0.363***	−0.351**
	(0.114)	(0.104)	(0.136)	(0.158)	(0.127)	(0.145)
Tanzania	−0.135***	−0.082**	−0.150***	−0.101*	−0.137***	−0.233***
	(0.036)	(0.040)	(0.041)	(0.059)	(0.038)	(0.037)
Uganda	−0.199***	−0.304***	−0.253***	0.063	−0.168**	−0.459***
	(0.072)	(0.084)	(0.075)	(0.135)	(0.085)	(0.082)
Zambia	0.545***	0.416***	0.512***	0.633***	0.635***	0.237***
	(0.043)	(0.059)	(0.048)	(0.072)	(0.045)	(0.041)
**Average**	**−0.004**	**0.043**	**−0.008**	**−0.001**	**0.079**	**−0.061**

Notes: Each column reports the coefficient in the regressions of ln (monthly earnings) on (a dummy for) a type of teachers with controls. The comparison group are other wage workers in columns 1–5 and other public sector employees in column 6. Controls include gender, age, age-squared, urbanicity and (a dummy for) post-secondary education. The top and bottom 1% earnings are trimmed. The survey in Malawi did not differentiate the levels of teachers. Sample is restricted to workers with secondary education or more. Standard errors in parentheses. *** p < 0.01, ** p < 0.05, * p < 0.1. *Source:* Surveys listed in Table 1.

**Table A5:** Hourly earnings differentials between teachers and other wage workers (trimmed sample).

	All teachers		Primary teachers	Secondary teachers	Public school teachers	Public school teachers relative to other public sector employees
	OLS	Median	OLS			
	(1)	(2)	(3)	(4)	(5)	(6)
Burkina Faso	0.598***	0.764***	0.619***	0.479**	0.670***	0.513***
	(0.095)	(0.093)	(0.101)	(0.217)	(0.103)	(0.123)
Côte d'Ivoire	0.840***	0.805***	0.808***	0.878***	1.079***	0.670***
	(0.113)	(0.126)	(0.148)	(0.161)	(0.133)	(0.127)
DR Congo	0.073**	0.123***	0.062	0.126*	0.047	0.057
	(0.036)	(0.037)	(0.039)	(0.076)	(0.039)	(0.040)
The Gambia	N.A.					

Ghana	0.239***	0.270***	0.217***	0.268***	0.338***	0.166***
	(0.046)	(0.053)	(0.058)	(0.066)	(0.052)	(0.052)
Liberia	−0.050	0.012	−0.032	−0.120	0.049	0.067
	(0.078)	(0.081)	(0.086)	(0.158)	(0.091)	(0.096)
Malawi	0.159**	0.190***	N.A.		0.243***	0.083
	(0.065)	(0.069)			(0.071)	(0.078)
Namibia	0.565***	0.527***	0.594***	0.514***	0.584***	0.396***
	(0.056)	(0.076)	(0.066)	(0.085)	(0.058)	(0.050)
Niger	0.124	0.081	0.154	0.031	0.218*	0.012
	(0.104)	(0.098)	(0.117)	(0.194)	(0.114)	(0.128)
Nigeria	−0.201***	−0.208***	−0.285***	−0.031	−0.001	−0.105
	(0.075)	(0.077)	(0.087)	(0.114)	(0.082)	(0.077)
Senegal	0.534***	0.553***	0.549***	0.513***	0.588***	0.393***
	(0.054)	(0.065)	(0.068)	(0.080)	(0.056)	(0.053)
Sierra Leone	0.154	0.124	0.230	0.012	0.154	0.175
	(0.145)	(0.130)	(0.168)	(0.216)	(0.165)	(0.196)
Tanzania	0.011	0.034	0.002	0.031	0.021	−0.126***
	(0.040)	(0.050)	(0.046)	(0.066)	(0.042)	(0.040)
Uganda	−0.053	−0.176*	−0.119	0.269*	−0.007	−0.253**
	(0.086)	(0.100)	(0.090)	(0.161)	(0.101)	(0.116)
Zambia	0.723***	0.546***	0.685***	0.823***	0.813***	0.369***
	(0.048)	(0.065)	(0.054)	(0.080)	(0.051)	(0.043)
**Average**	**0.265**	**0.260**	**0.268**	**0.292**	**0.343**	**0.173**

Notes: Each column reports the coefficient in the regressions of ln (monthly earnings) on (a dummy for) a type of teachers with controls. The comparison group are other wage workers in columns 1–5 and other public sector employees in column 6. Controls include gender, age, age-squared, urbanicity and (a dummy for) post-secondary education. The top and bottom 1% earnings are trimmed. The survey in Malawi did not differentiate the levels of teachers and the survey in The Gambia did not report weekly earnings. Sample is restricted to workers with secondary education or more. Standard errors in parentheses. *** p < 0.01, ** p < 0.05, * p < 0.1. *Source:* Surveys listed in Table 1.

**Table A6:** Earnings differentials between teachers and other wage workers by contract type (trimmed sample).

	Monthly		Hourly	
	Permanent	Fixed term/temporary	Permanent	Fixed term/temporary
	(1)	(2)	(3)	(4)
Burkina Faso	0.442***	0.030	0.620***	0.402
	(0.095)	(0.263)	(0.098)	(0.268)
Côte d'Ivoire	0.686***	0.020	1.090***	0.510***
	(0.125)	(0.146)	(0.140)	(0.162)
DR Congo	−0.048	−0.133***	0.113***	0.050
	(0.041)	(0.050)	(0.043)	(0.052)
The Gambia	N.A.			
Ghana	N.A.			
Liberia	N.A.			
Malawi	N.A.			
Namibia	0.389***	0.325***	0.561***	0.510***
	(0.055)	(0.111)	(0.060)	(0.120)
Niger	−0.042	−0.304**	0.191*	−0.087
	(0.091)	(0.128)	(0.099)	(0.141)
Nigeria	N.A.			
Senegal	0.197***	0.136	0.544***	0.384***
	(0.053)	(0.104)	(0.058)	(0.111)
Sierra Leone	−0.471***	−0.686**	0.081	0.534
	(0.119)	(0.287)	(0.151)	(0.414)
Tanzania	−0.138***	−0.158	0.013	−0.103
	(0.037)	(0.140)	(0.041)	(0.158)
Uganda	−0.187**	−0.206**	−0.008	−0.117
	(0.089)	(0.097)	(0.106)	(0.113)
Zambia	0.617***	0.031	0.793***	0.182
	(0.045)	(0.104)	(0.051)	(0.116)
**Average**	**0.145**	**−0.095**	**0.400**	**0.227**

Notes: Each column reports the coefficient in the regressions of ln (monthly/hourly earnings) on (a dummy for) a type of teachers with controls. The comparison group are other wage workers. Controls include gender, age, age-squared, urbanicity and (a dummy for) post-secondary education. The top and bottom 1% earnings are trimmed. Surveys in The Gambia, Ghana, Liberia, Malawi, and Nigeria did not report whether a worker was on a permanent or fixed term/temporary contract. Sample is restricted to workers with secondary education or more. Standard errors in parentheses. *** p < 0.01, ** p < 0.05, * p < 0.1. *Source:* Surveys listed in Table 1.

**Table A7:** Median regression estimates of monthly earning differentials (trimmed sample).

	All teachers	Primary teachers	Secondary teachers	Public school teachers	Public school teachers relative to other public sector employees
	(1)	(2)	(3)	(4)	(5)
Burkina Faso	0.629***	0.635***	0.449**	0.706***	0.456***
	(0.088)	(0.094)	(0.201)	(0.094)	(0.101)
Côte d'Ivoire	0.475***	0.528***	0.170	0.686***	0.333***
	(0.116)	(0.154)	(0.164)	(0.131)	(0.106)
DR Congo	−0.090***	−0.083***	−0.115**	−0.092***	−0.029*
	(0.027)	(0.029)	(0.057)	(0.028)	(0.017)
The Gambia	−0.149***	−0.304***	0.060	−0.110*	−0.092
	(0.050)	(0.059)	(0.070)	(0.058)	(0.056)
Ghana	−0.023	−0.068	0.053	0.032	−0.142***
	(0.045)	(0.054)	(0.063)	(0.050)	(0.042)
Liberia	−0.281***	−0.238***	−0.425**	−0.154	−0.112
	(0.080)	(0.089)	(0.165)	(0.096)	(0.089)
Malawi	0.140**	–	–	0.228***	0.075
	(0.057)	–	–	(0.063)	(0.054)
Namibia	0.359***	0.354***	0.372***	0.373***	0.256***
	(0.071)	(0.085)	(0.108)	(0.073)	(0.056)
Niger	−0.168*	−0.064	−0.347**	−0.033	−0.160**
	(0.092)	(0.103)	(0.170)	(0.098)	(0.079)
Nigeria	−0.297***	−0.355***	−0.134	−0.068	−0.115
	(0.072)	(0.081)	(0.108)	(0.077)	(0.074)
Senegal	0.225***	0.266***	0.079	0.228***	0.059
	(0.058)	(0.073)	(0.086)	(0.060)	(0.050)
Sierra Leone	−0.201*	−0.203	−0.188	−0.153	−0.166
	(0.104)	(0.128)	(0.150)	(0.113)	(0.107)
Tanzania	−0.082**	−0.105**	−0.037	−0.075*	−0.184***
	(0.040)	(0.046)	(0.066)	(0.042)	(0.043)
Uganda	−0.304***	−0.350***	0.018	−0.270***	−0.505***
	(0.084)	(0.088)	(0.158)	(0.101)	(0.085)
Zambia	0.416***	0.425***	0.412***	0.458***	0.146***
	(0.059)	(0.066)	(0.099)	(0.062)	(0.034)

Notes: Each column reports the coefficient in the median regressions of ln (monthly earnings) on (a dummy for) a type of teachers with controls. The comparison group are other wage workers in columns 1–5 and other public sector employees in column 6. Controls include gender, age, age-squared, urbanicity and (a dummy for) post-secondary education. The top and bottom 1% earnings are trimmed. The survey in Malawi did not differentiate the levels of teachers. Sample is restricted to workers with secondary education or more. Standard errors in parentheses. *** p < 0.01, ** p < 0.05, * p < 0.1. - indicates that data are not available. *Source:* Surveys listed in Table 1.

**Table A8:** Median regression estimates of hourly earning differentials (trimmed sample).

	All teachers	Primary teachers	Secondary teachers	Public school teachers	Public school teachers relative to other public sector employees
	(1)	(2)	(3)	(4)	(5)
Burkina Faso	0.764***	0.798***	0.539***	0.871***	0.648***
	(0.093)	(0.097)	(0.208)	(0.099)	(0.102)
Côte d'Ivoire	0.805***	0.808***	0.802***	1.041***	0.575***
	(0.126)	(0.170)	(0.184)	(0.141)	(0.133)
DR Congo	0.123***	0.119***	0.140*	0.098**	0.101***
	(0.037)	(0.040)	(0.078)	(0.039)	(0.035)
The Gambia	–	–	–	–	–
	–	–	–	–	–
Ghana	0.270***	0.246***	0.305***	0.314***	0.128**
	(0.053)	(0.065)	(0.074)	(0.060)	(0.056)
Liberia	0.012	0.047	−0.041	0.121	0.218**
	(0.081)	(0.088)	(0.162)	(0.096)	(0.088)
Malawi	0.190***	–	–	0.283***	0.168**
	(0.069)	–	–	(0.075)	(0.073)
Namibia	0.527***	0.563***	0.467***	0.525***	0.449***
	(0.076)	(0.090)	(0.115)	(0.079)	(0.059)
Niger	0.081	0.133	−0.022	0.155	−0.046
	(0.098)	(0.108)	(0.179)	(0.107)	(0.093)
Nigeria	−0.208***	−0.246***	−0.075	0.016	−0.078
	(0.077)	(0.092)	(0.122)	(0.080)	(0.080)
Senegal	0.553***	0.585***	0.519***	0.608***	0.349***
	(0.065)	(0.084)	(0.099)	(0.064)	(0.058)
Sierra Leone	0.124	0.146	0.092	0.033	0.173
	(0.130)	(0.152)	(0.196)	(0.146)	(0.148)
Tanzania	0.034	0.021	0.035	0.048	−0.075*
	(0.050)	(0.057)	(0.082)	(0.052)	(0.043)
Uganda	−0.176*	−0.187*	0.287	−0.166	−0.394***
	(0.100)	(0.105)	(0.188)	(0.119)	(0.115)
Zambia	0.546***	0.532***	0.568***	0.585***	0.299***
	(0.065)	(0.072)	(0.108)	(0.069)	(0.040)

Notes: Each column reports the coefficient in the median regressions of ln (hourly earnings) on (a dummy for) a type of teachers with controls. The comparison group are other wage workers in columns 1–5 and other public sector employees in column 6. Controls include gender, age, age-squared, urbanicity and (a dummy for) post-secondary education. The top and bottom 1% earnings are trimmed. The survey in Malawi did not differentiate the levels of teachers. Sample is restricted to workers with secondary education or more. Standard errors in parentheses. *** p < 0.01, ** p < 0.05, * p < 0.1. - indicates that data are not available. *Source:* Surveys listed in Table 1.

**Table A9:** Teacher age distribution.

Country	Mean	Standard deviation	25th percentile	Median	75th percentile
	(1)	(2)	(3)	(4)	(5)
Burkina Faso	35.1	7.5	30	34	39
Côte d'Ivoire	38.4	9.4	32	37	44
DR Congo	37.5	11.0	29	36	46
The Gambia	35.2	9.5	28	33	42
Ghana	35.0	10.6	27	32	42
Liberia	44.6	10.9	36	45	53
Malawi	38.2	9.6	31	38	44
Namibia	39.6	10.6	30	41	48
Niger	37.1	9.6	30	36	43
Nigeria	40.7	10.3	32	42	49
Senegal	37.1	8.7	30	35	42
Sierra Leone	41.8	9.3	35	42	49
Tanzania	38.6	10.3	30	36	46
Uganda	37.5	10.1	29	37	45
Zambia	35.3	7.8	30	34	39
**Average**	**38.1**	**9.7**	**30.6**	**37.2**	**44.7**

Notes: Teachers include survey respondents who self-identified as a primary or secondary school teacher and a wage worker. *Source:* Surveys listed in Table 1.

**Table A10:** Demographic characteristics of teachers by contract type.

	Permanent				Fixed term/temporary			
Country	Age	Percent male	Percent urban	Percent working in private schools	Age	Percent male	Percent urban	Percent working in private schools
	(1)	(2)	(3)	(4)	(1)	(2)	(3)	(4)
Burkina Faso	35.2	58.8%	76.9%	17.5%	34.0	48.6%	71.4%	45.7%
Côte d'Ivoire	39.7	78.7%	75.0%	11.1%	36.0	77.6%	77.6%	34.5%
DR Congo	37.9	72.0%	52.4%	12.6%	37.2	73.5%	47.3%	18.4%
The Gambia	–	–	–	–	–	–	–	–
Ghana	–	–	–	–	–	–	–	–
Liberia	–	–	–	–	–	–	–	–
Malawi	–	–	–	–	–	–	–	–
Namibia	40.4	33.9%	48.9%	4.9%	35.3	41.3%	39.7%	4.8%
Niger	39.3	54.1%	88.2%	15.7%	31.5	33.3%	91.7%	23.5%
Nigeria	–	–	–	–	–	–	–	–
Senegal	37.4	78.1%	84.5%	6.5%	35.0	70.4%	77.5%	29.6%
Sierra Leone	42.1	62.8%	79.4%	22.6%	39.8	66.7%	75%	45.8%
Tanzania	39.0	43.5%	85.3%	6.7%	32.5	52.4%	85.7%	4.8%
Uganda	41.3	67.0%	23.0%	1.0%	32.0	51.4%	38.6%	25.7%
Zambia	35.5	49.4%	58.6%	7.6%	34.0	51.6%	68.8%	42.2%
**Average**	**38.8**	**59.8%**	**67.2%**	**10.6%**	**34.7**	**56.7%**	**67.3%**	**27.5%**

Notes: Teachers include survey respondents who self-identified as a primary or secondary school teacher and a wage worker. Teachers were considered working in private schools unless they reported government (at any level) or any public entity as their employer. - indicates that data are not available to separate teachers by contract type. *Source:* Surveys listed in Table 1.

**Table A11:** Educational attainment of teachers by contract type.

	Permanent			Fixed term/temporary		
Country	Primary	Secondary	Post-Secondary	Primary	Secondary	Post-Secondary
	(1)	(2)	(3)	(4)	(5)	(6)
Burkina Faso	4.2	74.6	21.2	22.9	60.0	17.1
Côte d'Ivoire	0.1	55.5	44.4	1.2	57.1	41.7
DR Congo	2.3	82.4	15.4	3.3	83.1	13.6
The Gambia	N.A.					
Ghana	N.A.					
Liberia	N.A.					
Malawi	N.A.					
Namibia	1.0	27.7	71.3	1.6	46.0	52.4
Niger	5.9	67.1	27.1	0.0	69.4	30.6
Nigeria	N.A.					
Senegal	2.2	47.4	50.4	5.7	50.0	44.3
Sierra Leone	2.1	22.8	75.1	0.0	16.7	83.3
Tanzania	0.3	35.5	64.2	0.0	23.8	76.2
Uganda	0.0	2.0	98.0	1.5	15.0	83.5
Zambia	0.5	27.3	72.2	1.6	34.4	64.1
**Average**	**1.9**	**44.2**	**53.9**	**4.2**	**45.6**	**50.7**

Notes: Teachers include survey respondents who self-identified as a primary or secondary school teacher and a wage worker. The number in each cell represents the proportion of teachers with each level of education. Surveys in The Gambia, Ghana, Liberia, Malawi, and Nigeria did not report whether a worker was on a permanent or fixed term/temporary contract. *Source:* Surveys listed in Table 1.

**Table A12:** Average working hours and **median** earnings by contract type.

	Monthly earnings in PPP ($)		Monthly earnings relative to GDP per capita		Hourly earnings in PPP ($)		Hours worked weekly		Percent of having a second job	
	Permanent	Fixed term/temporary	Permanent	Fixed term/temporary	Permanent	Fixed term/temporary	Permanent	Fixed term/temporary	Permanent	Fixed term/ temporary
	(1)	(2)	(3)	(4)	(5)	(6)	(7)	(8)	(9)	(10)
Burkina Faso	650	232	4.8	1.7	4.0	1.5	39.7	33.8	21.2%	32.4%
Côte d'Ivoire	1,059	371	4.3	1.5	7.6	4.0	31.8	30.3	16.9%	14.1%
DR Congo	100	92	1.6	1.4	0.7	0.7	34.7	33.5	45.4%	50.1%
The Gambia	N.A.									
Ghana	N.A.									
Liberia	N.A.									
Malawi	N.A.									
Namibia	2,351	1,627	2.9	2.0	14.8	9.6	39.3	39.6	6.6%	4.8%
Niger	542	334	6.9	4.2	4.2	2.4	33.9	35.5	21.7%	0%
Nigeria	N.A.									
Senegal	832	690	4.7	3.9	5.9	4.5	34.6	38.5	13.7%	18.3%
Sierra Leone	334	278	2.3	1.9	2.6	4.0	31.7	30.9	20.9%	25.0%
Tanzania	805	713	3.8	3.4	5.0	4.0	42.1	46.9	22.7%	19.1%
Uganda	345	252	2.4	1.8	2.3	1.5	38.1	41.5	51.0%	41.4%
Zambia	1,691	1,431	5.3	4.4	11.2	8.3	39.1	40.4	10.9%	6.3%
**Average**	**871**	**593**	**3.9**	**2.6**	**5.8**	**4.1**	**36.5**	**37.1**	**23.1%**	**21.2%**

Notes: Teachers include survey respondents who self-identified as a primary or secondary school teacher and a wage worker. All the earnings are in PPP ($,2011). The top and bottom 1% earnings are trimmed. Sample only include workers with secondary or post-secondary education and those who reported both earnings and hours worked weekly. Surveys in The Gambia, Ghana, Liberia, Malawi, and Nigeria did not report whether a worker was on a permanent or fixed term/temporary contract. *Source:* Surveys listed in Table 1.

**Table A13:** Occupations of secondary job for teachers.

	Proportion of having a second job	Tutoring/teaching	Farming or animal breeding	Sales/vending	Other
	(1)	(2)	(3)	(4)	(5)
Burkina Faso	22.3%	5%	81%	5%	9%
Côte d'Ivoire	15.9%	12%	47%	22%	19%
DR Congo	46.9%	1%	50%	7%	42%
The Gambia	N.A.				
Ghana	26.9%	3%	73%	8%	16%
Liberia	5.8%	30%	40%	10%	20%
Malawi	3.3%	92%	0%	8%	0%
Namibia	6.5%	9%	32%	9%	50%
Niger	15.4%	6%	56%	11%	27%
Nigeria	2.1%	40%	20%	20%	20%
Senegal	14.8%	N.A.			
Sierra Leone	21.2%	4%	42%	16%	38%
Tanzania	22.5%	3%	74%	16%	7%
Uganda	47.1%	4%	65%	10%	21%
Zambia	10.1%	N.A.			
**Average**	**18.6%**	**17%**	**48%**	**12%**	**22%**

Notes: Teachers include survey respondents who self-identified as a primary or secondary school teacher and a wage worker. The first column reports the proportion of teachers who reported to hold a secondary job. Columns 2 – 5 report the distribution of occupations in the secondary job conditional on having a secondary job. The survey in The Gambia did not report whether a worker held a secondary job. The surveys in Senegal and Zambia did not report the occupation for the secondary job. *Source:* Surveys listed in Table 1.

**Table A14:** Occupations of secondary job for teachers by contract type.

	Permanent					Fixed-term/Temporary				
	Proportion of having a second job	Tutoring/teaching	Farming or animal breeding	Sales/vending	Other	Proportion of having a second job	Tutoring/teaching	Farming or animal breeding	Sales/ vending	Other
	(1)	(2)	(3)	(4)	(5)	(6)	(7)	(8)	(9)	(10)
Burkina Faso	21.2%	6%	81%	3%	10%	32.4%	0%	83%	8%	9%
Côte d'Ivoire	16.9%	17%	43%	13%	27%	14.1%	0%	50%	41%	9%
DR Congo	45.4%	1%	49%	6%	44%	50.1%	1%	52%	9%	38%
The Gambia	N.A.					N.A.				
Ghana	N.A.					N.A.				
Liberia	N.A.					N.A.				
Malawi	N.A.					N.A.				
Namibia	6.6%	11%	26%	11%	52%	4.8%	0%	67%	0%	33%
Niger	21.7%	6%	56%	11%	27%	0%	0%	0%	0%	0%
Nigeria	N.A.					N.A.				
Senegal	N.A.					N.A.				
Sierra Leone	13.7%	4%	41%	10%	45%	18.3%	0%	40%	0%	60%
Tanzania	20.9%	4%	76%	10%	10%	25.0%	0%	40%	40%	20%
Uganda	22.7%	2%	71%	8%	19%	19.1%	7%	55%	14%	24%
Zambia	51.0%	N.A.				41.4%	N.A.			
**Average**	24.5%	6%	55%	9%	29%	22.8%	1%	55%	16%	28%

Notes: Teachers include survey respondents who self-identified as a primary or secondary school teacher and a wage worker. The first and sixth column reports the proportion of teachers who reported to hold a secondary job. Columns 2 – 5 and 7–10 report the distribution of occupations in the secondary job conditional on having a secondary job. Surveys in The Gambia, Ghana, Liberia, Malawi, and Nigeria did not report whether a worker was on a permanent or fixed term/temporary contract. The surveys in Senegal and Zambia did not report the occupation for the secondary job. *Source:* Surveys listed in Table 1.

**Table A15:** Median earnings by teacher type.

	Monthly earnings				Hourly earnings			
	Teachers	Public School	Primary	Secondary	Teachers	Public School	Primary	Secondary
	(1)	(2)	(3)	(4)	(5)	(6)	(7)	(8)
Burkina Faso	650	682	617	773	4.0	4.4	3.9	4.8
Côte d'Ivoire	871	1102	975	733	6.6	7.6	6.9	5.3
DR Congo	100	100	98	102	0.7	0.7	0.7	0.8
The Gambia	265	269	209	327	0.0	–	–	–
Ghana	656	667	500	778	4.4	4.8	3.7	5.6
Liberia	220	220	220	237	1.7	1.8	1.7	1.7
Malawi	337	353	–	–	2.6	3.0	0.0	0.0
Namibia	2,306	2,315	1,989	2,685	14.7	14.7	13.6	16.7
Niger	401	406	360	700	2.8	3.1	2.6	6.7
Nigeria	460	529	402	575	2.9	3.4	2.5	3.6
Senegal	819	819	681	862	5.6	5.9	5.1	7.1
Sierra Leone	334	353	334	362	2.8	2.6	2.8	2.5
Tanzania	805	805	724	934	5.0	5.0	4.5	5.7
Uganda	321	346	321	375	2.1	2.3	2.0	2.8
Zambia	1,639	1,729	1,610	1,807	10.7	11.2	10.2	11.7
**Average**	**679**	**713**	**646**	**803**	**4.4**	**5.0**	**4.3**	**5.4**

Notes: All the earnings are in PPP ($,2011). Teachers include survey respondents who self-identified as a primary or secondary school teacher and a wage worker. Sample only include teachers with secondary or post-secondary education. The top and bottom 1% earnings are trimmed. - indicates that data are not available to distinguish teachers across the indicated categories. *Source:* Surveys listed in Table 1.

**Table A16:** Monthly median earnings for workers with secondary school education by economic activity.

		Other workers with secondary school education								
Country	Teachers	Agriculture	Mining	Manufacturing	Construction	Retail	Financial services	Admin services	Health and social services	Public administration
	(1)	(2)	(3)	(4)	(5)	(6)	(7)	(8)	(9)	(10)
Burkina Faso	603	*209*	186	232	232	162	*492*	255	278	320
Côte d'Ivoire	852	265	*311*	778	335	786	*713*	540	481	562
DR Congo	96	65	363	136	109	114	241	91	82	97
The Gambia	254	204	*210*	179	270	189	408	*194*	240	236
Ghana	278	278	500	228	444	222	278	278	364	667
Liberia	207	255	370	171	278	143	417	222	226	185
Malawi	258	176	*338*	191	206	147	559	287	221	268
Namibia	1,447	334	1,085	506	506	362	1,356	362	687	1,085
Niger	338	273	1,763	267	*383*	240	*389*	*244*	420	540
Nigeria	230	207	*589*	402	460	155	*195*	–	270	352
Senegal	688	431	791	431	388	302	735	–	485	647
Sierra Leone	301	*389*	565	222	556	222	425	334	334	289
Tanzania	724	402	1,073	547	580	560	668	410	672	731
Uganda	188	148	*987*	178	237	158	*99*	*420*	*271*	*346*
Zambia	1,610	215	1,073	394	429	286	1,594	286	1,073	1,252
**Average**	**538**	**257**	**680**	**324**	**361**	**270**	**571**	**302**	**407**	**505**

Notes: All the earnings are in PPP ($,2011). Teachers include survey respondents who self-identified as a primary or secondary school teacher and a wage worker. Other wage workers include respondents who self-identified as a wage worker in other corresponding occupation groups; university faculty, special education teachers and other teachers were excluded from the group of other wage workers. ISIC Rev.4 are used to classify economic activities (United Nations Statistics Division, 2007). Earnings in italics are estimated based on fewer than 10 observations. Sample only includes workers with secondary or post-secondary education. The top and bottom 1% earnings are trimmed. - indicates no observations in those categories. Source: Surveys listed in Table 1.

**Table A17:** Monthly median earnings for workers with post-secondary education by economic activity.

		Other workers with post-secondary education								
Country	Teachers	Agriculture	Mining	Manufacturing	Construction	Retail	Financial services	Admin services	Health and social services	Public administration
	(1)	(2)	(3)	(4)	(5)	(6)	(7)	(8)	(9)	(10)
Burkina Faso	756	*251*	*1,123*	*394*	*251*	162	200	464	972	603
Côte d'Ivoire	889	*1,716*	*1,025*	*1,059*	*720*	*501*	*979*	*678*	*953*	969
DR Congo	109	172	452	167	608	163	376	330	109	136
The Gambia	286	514	–	459	*214*	255	510	–	373	306
Ghana	778	1,000	1,444	556	722	444	1,067	611	861	889
Liberia	231	895	*2,775*	*740*	*648*	*1,471*	*463*	393	472	*531*
Malawi	330	432	*471*	618	430	662	1,029	735	751	801
Namibia	2,532	1,899	3,436	1,808	1,989	1,627	2,078	1,808	2,351	1,718
Niger	374	*1,511*	*3,111*	*3,511*	*1,422*	*893*	*1,111*	*578*	*1,244*	1,244
Nigeria	483	667	*1,853*	*977*	920	*546*	575	–	737	805
Senegal	862	1,040	981	1,078	862	647	970	–	862	1,121
Sierra Leone	350	*2,948*	*541*	*311*	*222*	*528*	667	434	378	834
Tanzania	831	*895*	*1,207*	1,127	1,288	740	1,368	966	1,046	1,288
Uganda	336	*168*	*987*	*869*	449	*395*	*622*	444	465	464
Zambia	1,717	1,503	2,147	1,073	1,073	1,073	2,111	1,556	1,789	1,700
**Average**	**724**	**1,041**	**1,540**	**983**	**788**	**674**	**942**	**750**	**891**	**894**

Notes: All the earnings are in PPP ($,2011). Teachers include survey respondents who self-identified as a primary or secondary school teacher and a wage worker. Other wage workers include respondents who self-identified as a wage worker in other corresponding occupation groups; university faculty, special education teachers and other teachers were excluded from the group of other wage workers. ISIC Rev.4 are used to classify economic activities (United Nations Statistics Division, 2007). Earnings in italics are estimated based on fewer than 10 observations. Sample only includes workers with secondary or post-secondary education. The top and bottom 1% earnings are trimmed. - indicates no observations in those categories. *Source:* Surveys listed in Table 1.

**Table A18:** Monthly earnings structure (R-squared, untrimmed sample).

	Teachers			Other wage workers
	Teacher	Primary teacher	Secondary teacher	
	(1)	(2)	(3)	(4)
Burkina Faso	0.03	0.039	0.058	0.07
Côte d'Ivoire	0.184	0.117	0.348	0.217
DR Congo	0.124	0.109	0.26	0.186
The Gambia	0.188	0.185	0.063	0.176
Ghana	0.312	0.299	0.304	0.232
Liberia	0.133	0.138	0.204	0.14
Malawi	0.206	N.A.		0.353
Namibia	0.203	0.236	0.13	0.311
Niger	0.134	0.101	0.601	0.261
Nigeria	0.265	0.23	0.355	0.312
Senegal	0.085	0.066	0.14	0.364
Sierra Leone	0.143	0.13	0.169	0.124
Tanzania	0.175	0.241	0.193	0.289
Uganda	0.177	0.27	0.101	0.356
Zambia	0.164	0.199	0.152	0.365
**Average**	**0.168**	**0.169**	**0.220**	**0.250**

Notes: Each cell reports the R-squared of the regression of ln (monthly earnings) on gender, age, age-squared, urbanicity and (a dummy for) post-secondary education for a specified occupation group. Sample includes wage workers with secondary or post-secondary education. The survey in Malawi did not differentiate the levels of teachers. *Source:* Surveys listed in Table 1.

**Table A19:** Percent of workers receiving employee benefits.

	Paid leave			Medical benefits			Social security/Pension	
	Teachers	Other workers		Teachers	Other workers		Teachers	Other workers
	(1)	(2)		(3)	(4)		(5)	(6)
Burkina Faso	81.82	50.05		2.80	18.18		81.56	48.43
Côte d'Ivoire	60.02	43.85		31.94	36.48		N.A.	
DR Congo	11.03	11.49		1.62	7.16		4.18	10.37
The Gambia	60.62	52.59		N.A.			65.93	51.35
Ghana	89.17	58.03		23.89	29.46		78.78	37.56
Liberia	N.A.			26.14	30.18		N.A.	
Malawi	N.A.							
Namibia	92.36	82.65		78.87	40.04		74.08	41.15
Niger	78.57	60.69		35.71	42.48		48.21	47.80
Nigeria	N.A.			5.11	20.92		52.12	40.69
Senegal	N.A.			32.35	19.95		61.76	46.31
Sierra Leone	N.A.			N.A.			N.A.	
Tanzania	84.05	59.25		N.A.			95.17	70.88
Uganda	55.15	27.82		4.85	17.93		42.42	23.45
Zambia	91.88	55.35		N.A.			98.72	69.92
**Average**	**70.47**	**50.18**		**24.33**	**26.28**		**63.90**	**44.36**

Notes: Teachers include survey respondents who self-identified as a primary or secondary school teacher and a wage worker. Other wage workers include respondents who self-identified as a wage worker in other occupations; university faculty, special education teachers and other teachers were excluded from the group of other wage workers. Sample only includes teachers and other wage workers with secondary or post-secondary education. *Source:* Surveys listed in Table 1.

**Table A20:** Percent of teachers receiving employee benefits by contract type.

	Paid leave		Medical benefits		Social security/Pension	
	Permanent	Fixed term/ temporary	Permanent	Fixed term/ temporary	Permanent	Fixed term/ temporary
	(1)	(2)	(3)	(4)	(5)	(6)
Burkina Faso	85.32	42.31	2.72	3.70	85.03	42.31
Côte d'Ivoire	68.82	46.67	40.86	16.67	N.A.	
DR Congo	12.91	8.74	1.71	1.49	4.82	3.41
The Gambia	N.A.					
Ghana	N.A.					
Liberia	N.A.					
Malawi	N.A.					
Namibia	93.36	86.54	83.55	53.23	79.61	43.55
Niger	83.33	67.65	41.03	23.53	62.82	14.71
Nigeria	N.A.					
Senegal	N.A.		33.77	23.64	66.23	34.55
Sierra Leone	N.A.					
Tanzania	85.83	52.38	N.A.		96.77	66.67
Uganda	73.74	27.27	4.04	6.06	49.49	31.82
Zambia	94.51	68.00	N.A.		100.00	88.00
**Average**	**74.73**	**49.95**	**29.67**	**18.33**	**68.10**	**40.63**

Notes: Teachers include survey respondents who self-identified as a primary or secondary school teacher and a wage worker. Sample includes teachers with secondary or post-secondary education. Surveys in The Gambia, Ghana, Liberia, Malawi, and Nigeria did not report whether a worker was on a permanent or fixed term/temporary contract. *Source:* Surveys listed in Table 1.

**Table A21:** Proportion of teachers and other work receiving at least one benefit and the association between benefits and earnings.

	At least one benefit (%)			Monthly earnings		Hourly earnings	
	Teachers	Other workers	Ratio	Teachers	Other workers	Teachers	Other workers
	(1)	(2)	(3)	(4)	(5)	(6)	(7)
Burkina Faso	83.8	56.63	1.480	0.030*	0.048***	0.019	0.047***
				(0.016)	(0.011)	(0.016)	(0.011)
Cote d'Ivoire	64.58	53.06	1.217	0.212***	0.113***	0.127**	0.097***
				(0.056)	(0.009)	(0.050)	(0.009)
DR Congo	14.64	19.28	0.759	0.075***	0.116***	0.080***	0.090***
				(0.016)	(0.007)	(0.016)	(0.007)
The Gambia	72.57	57.45	1.263	0.143***	0.123***	N.A.	
				(0.051)	(0.018)		
Ghana	94.07	62.19	1.513	0.018*	0.099***	0.022**	0.076***
				(0.011)	(0.010)	(0.011)	(0.009)
Liberia	26.14	30.18	0.866	0.074	0.157***	0.046	0.123***
				(0.058)	(0.020)	(0.053)	(0.019)
Malawi	N.A.						
Namibia	96.34	82.82	1.163	0.072***	0.113***	0.069***	0.104***
				(0.013)	(0.007)	(0.013)	(0.006)
Niger	81.25	62.54	1.299	0.001	0.077***	−0.021	0.077***
				(0.037)	(0.017)	(0.036)	(0.016)
Nigeria	52.12	45.03	1.157	0.169***	0.125***	0.112***	0.087***
				(0.035)	(0.018)	(0.030)	(0.015)
Senegal	70.22	50.18	1.399	0.182***	0.187***	0.166***	0.169***
				(0.040)	(0.013)	(0.036)	(0.012)
Sierra Leone	N.A.						
Tanzania	97.47	75.01	1.299	0.059***	0.088***	0.052***	0.103***
				(0.020)	(0.014)	(0.018)	(0.012)
Uganda	68.48	36.55	1.874	0.196***	0.156***	0.147***	0.105***
				(0.064)	(0.027)	(0.044)	(0.025)
Zambia	98.72	73.23	1.348	0.057***	0.207***	0.050***	0.191***
				(0.010)	(0.009)	(0.009)	(0.008)

Notes: Columns 1–2 report the percent of workers who had access to at least one work benefit listed in Table A19, Table A20. Column 3 reports the ratio of the proportion of teachers receiving at least one benefit to that of other wage workers. Surveys in Malawi and Sierra Leone did not report work benefits. Columns 4–6 report the coefficients in the regressions of (a dummy for) at least having one benefit on ln (monthly or hourly earnings) with controls for teacher or other workers. Sample is restricted to workers with secondary education or more. Standard errors in parentheses. *** p < 0.01, ** p < 0.05, * p < 0.1. The survey in The Gambia did not report hourly earnings. *Source:* Surveys listed in Table 1.

**Table A22:** Proportion of teachers receiving “fringe benefits” or “social benefits, allowances and/or bonuses”.

Panel A: All grade 2 and grade 6 teachers										
	Benin	Burkina Faso	Burundi	Cameroon	Congo Rep.	Côte d'Ivoire	Niger	Senegal	Tchad	Togo
**Fringe benefits**										
Housing	**0.37**	0.24	**0.47**	0.30	0.14	**0.59**	0.20	0.19	0.22	0.05
Catering	0.01	0.06	0.00	0.04	0.06	0.05	0.02	0.06	0.02	0.01
Access to drinking water	0.17	0.29	0.03	0.16	0.10	0.27	0.17	**0.42**	0.11	0.13
Transport	0.07	0.01	0.22	0.03	**0.39**	0.21	0.12	0.04	0.20	0.02
**Social benefits, allowances and/or bonuses**										
Health insurance	0.03	0.03	0.11	0.01	0.01	0.11	0.03	0.11	0.01	**0.43**
Mutual health insurance	0.02	0.00	**0.87**	0.00	0.06	**0.53**	0.01	**0.53**	0.01	0.01
Pension or annuity plan	**0.48**	**0.34**	**0.46**	**0.31**	0.26	**0.59**	0.29	**0.40**	0.13	**0.40**
Housing allowance	**0.54**	**0.41**	**0.80**	**0.46**	0.00	**0.67**	**0.31**	**0.35**	0.26	0.03
Supervised study allowance	0.06	0.00	0.00	0.02	0.04	**0.38**	0.01	0.04	0.03	0.01
Examination allowance	**0.34**	0.23	0.00	0.23	0.12	0.23	0.10	**0.49**	**0.57**	0.19
Teacher's bonus	**0.45**	0.06	0.28	**0.48**	0.19	0.02	**0.48**	0.25	0.11	0.27
Back-to-school bonus	**0.66**	0.01	0.01	0.03	0.00	0.00	0.01	0.00	0.01	**0.46**
Assignment grant	0.10	0.03	0.00	0.02	0.12	0.00	0.06	0.05	0.10	0.01
Research allowance	0.01	0.01	0.00	**0.48**	0.26	0.00	0.01	**0.59**	0.26	**0.38**
Notes: Numbers greater than 0.3 are in bold to improve readability. *Source:*PASEC, 2015.										

Panel B: Grade 2 and grade 6 teachers: Civil servants only										
	Benin	Burkina Faso	Burundi	Cameroon	Congo Rep.	Côte d'Ivoire	Niger	Senegal	Tchad	Togo
	(1)	(2)	(3)	(4)	(5)	(6)	(7)	(8)	(9)	(10)
**Fringe benefits**										
Housing	**0.59**	0.17	**0.46**	**0.39**	0.18	**0.72**	**0.44**	**0.37**	**0.72**	0.04
Catering	0.01	0.00	0.00	0.04	0.00	0.03	0.00	0.01	0.04	0.02
Access to drinking water	0.15	0.25	0.03	0.23	0.07	**0.32**	0.20	0.28	0.23	0.11
Transport	0.10	0.00	0.22	0.00	**0.8**3	0.27	**0.32**	0.02	**0.64**	0.04
**Social benefits, allowances and/or bonuses**										
Health insurance	0.04	0.02	0.11	0.08	0.02	0.14	0.03	0.16	0.01	**0.79**
Mutual health insurance	0.00	0.00	**0.87**	0.00	0.09	**0.70**	0.00	**0.58**	0.00	0.02
Pension or annuity plan	**0.76**	**0.60**	**0.47**	**0.65**	0.62	**0.77**	**0.57**	**0.80**	**0.38**	**0.65**
Housing allowance	**0.90**	**0.71**	**0.80**	**0.85**	0.00	**0.88**	**0.83**	**0.93**	**0.86**	0.03
Supervised study allowance	0.03	0.00	0.00	0.04	0.02	**0.51**	0.02	0.01	0.08	0.02
Examination allowance	**0.50**	**0.43**	0.00	**0.53**	0.03	0.29	0.08	**0.74**	**0.81**	**0.33**
Teacher's bonus	**0.67**	0.14	0.28	**0.89**	**0.38**	0.02	**0.69**	**0.60**	**0.36**	**0.50**
Back-to-school bonus	**1.00**	0.01	0.01	0.01	0.00	0.00	0.00	0.00	0.02	**0.81**
Assignment grant	0.22	0.01	0.00	0.10	**0.31**	0.00	0.08	0.14	0.32	0.02
Research allowance	0.02	0.00	0.00	**0.85**	**0.75**	0.00	0.00	**0.84**	**0.86**	**0.63**

Notes: Numbers greater than 0.3 are in bold to improve readability. *Source:*PASEC, 2015.

**Table A23:** Heterogeneity across countries in economic indicators, and correlations with earnings premia.

	GDP per capita	Share of wage work in labor force (%)	Female labor force participation (%)	Earnings premium: Monthly	Earnings premium: Hourly
	(1)	(2)	(3)	(4)	(5)
Burkina Faso	1583	10	68	0.433	0.631
Côte d’Ivoire	2880	17	91	0.287	0.734
DR Congo	653	17	63	−0.121	0.043
Ghana	3807	15	79	0.051	0.324
Liberia	805	16	75	−0.475	−0.059
Malawi	1033	17	84	0.018	0.169
Namibia	9256	45	75	0.381	0.563
Niger	900	14	66	−0.156	0.096
Nigeria	5671	14	57	−0.250	−0.146
Senegal	2184	12	41	0.247	0.564
Sierra Leone	1692	12	65	−0.586	0.047
Tanzania	1423	12	87	−0.143	0.003
The Gambia	2091	18	64	−0.184	N.A.
Uganda	1637	18	70	−0.188	−0.027
Zambia	3633	24	61	0.522	0.697
Max	9,256	45	91	0.52	0.73
Min	653	10	41.3	−0.59	−0.15
Ratio: Max/Min	14.2	4.5	2.2	−0.9	−5.0
**Correlations**					
Monthly premium	0.40	0.39	−0.01	1.00	0.90
Hourly premium	0.32	0.32	0.00	0.90	1.00

Notes: GDP per capita, PPP (constant 2011 international $). *Source*: World Development Indicators (2017). Monthly and hourly earnings premia were from Table 8. Other statistics were derived from the surveys listed in Table 1.

**Table A24:** Heterogeneity across countries in indicators of the education sector, and correlations with earnings premia.

	Average years of schooling of teachers	Average years of schooling of teachers / other workers	% at least 1 benefit teachers/other workers	Teacher unionization (%)	Private school enrollments in 2014 (%)	Teacher share of labor force (earliest, latest) %	Avg. annual growth rate in teacher/labor force ratio over period (%)	Year of free primary education	Earning premium: Monthly	Earnings premium: Hourly
	(1)	(2)	(3)	(4)	(5)	(6)	(7)	(8)	(9)	(10)
Burkina Faso	13.1	1.07	1.48	73.8	22.6	0.8	6.3	2007	0.433	0.631
Côte d’Ivoire	13.9	1.09	1.22	35.8	25.1	1.0	2.5	2015	0.287	0.734
DR Congo	12.2	0.98	0.76	51.5	14.0	1.3	5.2	2019	−0.121	0.043
Ghana	14.7	1.22	1.51	N.A.	20.7	1.1	1.8	2005	0.051	0.324
Liberia	12.5	1.09	0.87	N.A.	41.1	1.3	1.4	2001	−0.475	−0.059
Malawi	14.2	1.11	N.A.	N.A.	2.5	0.9	3.1	1994	0.018	0.169
Namibia	15.3	1.30	1.16	N.A.	N.A.	2.2	0.0	2013	0.381	0.563
Niger	12.1	1.05	1.30	N.A.	6.2	0.7	7.0	2009	−0.156	0.096
Nigeria	15.1	1.10	1.16	N.A.	16.1	1.1	−0.1	1999	−0.250	−0.146
Senegal	13.7	1.19	1.40	N.A.	N.A.	1.1	3.4	2001	0.247	0.564
Sierra Leone	13.8	1.11	N.A.	53.2	N.A.	1.4	4.7	2018	−0.586	0.047
Tanzania	15.2	1.10	1.30	89.9	5.9	0.7	1.1	2001	−0.143	0.003
The Gambia	15.9	1.00	1.26	N.A.	N.A.	1.3	0.6	2013	−0.184	N.A.
Uganda	12.7	0.93	1.87	N.A.	N.A.	1.4	0.1	1997	−0.188	−0.027
Zambia	13.5	1.26	1.35	86.4	N.A.	1.0	2.7	2001	0.522	0.697
Max	15.9	1.30	1.87	89.9	41.14	2.23	7.0	2019	0.52	0.73
Min	12.1	0.93	0.76	35.8	2.5	0.67	−0.1	1994	−0.59	−0.15
Ratio: Highest/Lowest	1.3	1.40	2.47	2.5	16.5	3.4	−79.4	1.0	−0.9	−5.0
**Correlations**										
Monthly premium	0.10	0.53**	0.27	0.25	−0.11	−0.05	0.08	−0.06	1.00	0.90
Hourly premium	0.13	0.57**	0.19	−0.06	0.19	0.02	0.17	0.20	0.90	1.00

Notes: Average years of schooling was calculated based on the highest degree completed reported by survey respondents for both teachers and other wage workers. Reported work benefits for each country were listed in Table A19, Table A20. Surveys in Malawi and Sierra Leone did not report work benefits. Only six countries reported union status. Private school enrollments include both primary and secondary enrollments. For change in teacher/labor force ratio, period is: 2000–2010 for Nigeria; 2000–2017 for Liberia, Niger, Uganda, Zambia; 2000–2018 for Burkina Faso, Côte d’Ivoire, Namibia, Senegal, Gambia; 2000–2019 for Ghana; 2001–2018 for Sierra Leone, Tanzania; 2002–2015 for DRC; 2007–2018 for Malawi. *Sources:* Private enrollments and teacher numbers: UNESCO Institute for Statistics Education Statistics. Retrieved from: https://data.uis.unesco.org/. Labor Force: International Labor Organization. Retrieved from https://ilostat.ilo.org/. Year of Free Primary Education: Burkina Faso, Niger, Nigeria, and Senegal: Sandefur and Glassman (2015). Malawi, Tanzania, Uganda, and Zambia: Riddell (2003). Ghana and confirmation of Malawi: World Bank (2019b). The Gambia: UNESCO 2015. DRC: Latif and Adelman (2021). Sierra Leone: Samanova et al. (2021). Liberia: Waydon et al. (2016). Namibia: Iipinge and Likando (2013). Côte d’Ivoire: Oyeniran (2017). Monthly and hourly earnings premia were from Table 8. Other statistics were derived from the surveys listed in Table 1.

## Footnotes

1 See, for example, reporting on DRC (Bujakera, 2021), Kenya (Nyamai, 2021), Lesotho (Motsoeli, 2019), Mozambique (da Conceição, 2017), Uganda (Ahimbisibwe, 2019), and Zimbabwe (New Zimbabwe, 2022).

2 A question that is separate from the level of teacher salaries but equally important is the structure of teacher salaries. A range of recent work explores different teacher and other public sector pay structures in low- and middle-income countries (Barrera-Osorio & Raju, 2017; Cilliers, Kasirye, Leaver, Serneels, & Zeitlin, 2018; Gilligan, Karachiwalla, Kasirye, Lucas, & Neal, 2022; Leaver, Ozier, Serneels, & Zeitlin, 2021; Mbiti et al., 2019; Muralidharan & Sundararaman, 2011). Other aspects of teacher pay may also matter, such as the timeliness of payment (Kontagora, Watts, & Allsop, 2018).

3 Specifically, these statistics are provided by Pôle de Dakar, the Africa-based office of UNESCO’s International Institute for Educational Planning.

4 One recent report examines teacher pay across 13 countries in Sub-Saharan Africa and finds that primary school teachers are underpaid by the hour, compared to other professionals, and secondary school teachers are comparably paid. That analysis is pooled for the 13 countries, with no indication of country-by-country variation (see Bashir et al., 2018, Figure 4.7). Furthermore, that analysis is restricted to teachers and other workers with tertiary education, whereas we find that only about half of teachers across countries have post-secondary education (see the discussion in Section 3.1).

5 We use the World Bank 2019–2020 income and regional classifications (World Bank, 2019a).

6 The labor force is defined as people aged between 15 and 64 who worked, looked for a job in the past week, or had a job to return to.

7 The statistic of 2.2 percent for the US is derived from the May 2018 National Occupational Employment and Wage Estimates reported by the U.S. Bureau of Labor Statistics (2018). It is the share of all employment that is categorized as having the occupations of “Elementary and Middle School Teachers” or “Secondary School Teachers.”

8 Formal sector employees are people in the labor force who reported they earned a wage or salary in their primary jobs.

9 PASEC stands for Programme d’analyse des systèmes éducatifs de la Confemen.

10 Other factors affect whether teachers (and other workers) acquire a second job, including spousal occupation and earnings (Goux, Maurin, & Petrongolo, 2014). While a full exploration of these factors is beyond what the data we use will permit (as spousal relationships are not always clear), it merits exploration in future work.

11 While this resembles the model of wage determination proposed by Mincer (1974), we employ it here with a descriptive rather than a causal interpretation.

12 Appendix Table A2 shows the proportion of respondents who reported in-kind payments (median is 10 percent for teachers and 18 percent for other workers) and – for those who did – how much of their income they reported as such (median is 20 percent for teachers and 32 percent for other workers).

13 This could affect our results if the reported earnings in surveys from those countries did not include in-kind payment, and teachers in those countries were systematically more (or less) likely to receive in-kind payment relative to employees in other professions. Appendix Table A3 reports regression estimates using monthly earnings excluding the in-kind payments for countries that report in-kind payments. The results are consistent with estimates using earnings with in-kind payments in our main analysis.

14 Note that in our analysis of the characteristics of teachers we include teachers with all levels of education, and report results separately by level of education. For comparisons of earnings, we also restrict the sample to wage workers with secondary or more education.

15 Our main estimates of earning premia are similar to those for the trimmed sample or those using median regression. Estimates for these models are reported in Appendix Table A4, Table A5, Table A6, Table A7, Table A8.

16 We exclude these “other teachers” from both the group of teachers and from the comparator group of “other wage workers.”.

17 In this section and the next (3.1 and 3.2), in order to fully describe the teacher labor force, we include teachers who have only a primary education.

18 We present more detail on the age distribution of teachers in Appendix Table A9. Some countries, like Burkina Faso, have relatively low variation in teacher age; others, like DR Congo, have higher variation. The cross-country average of the 25th percentile in teacher age is 31 years old, and the average 75th percentile in teacher age is 45 years old.

19 Data from World Development Indicators (World Bank, 2021).

20 The surveys only collected the area an individual lived but not necessarily where the person worked, which would be the ideal way to distinguish whether teachers both lived and worked in urban areas or lived in urban areas but traveled to work in rural areas. The latter is not uncommon in many African countries, which means that our data are not ideal for analyzing urban–rural differences in teacher labor markets. Furthermore, many countries have centralized allocation of teachers rather than local hiring.

21 Teachers were considered working in private schools unless they reported government (at any level) or any public entity as their employer.

22 Many countries employ part of their teaching force on short-term, untenured contracts. Research in Kenya and India shows higher test scores for students taught by locally hired teachers on annual contracts (Duflo, Dupas, & Kremer, 2015; Muralidharan & Sundararaman, 2013), although in Kenya, those gains disappeared when the contracts were administered by government rather than by non-government organizations (Bold, Kimenyi, Mwabu, & Sandefur, 2018).

23 Beyond the share of teachers working at each level of education, we compare the characteristics of teachers on a permanent contract versus on fixed term or temporary contracts in Appendix Table A10, Table A11, Table A12.

24 There are far more missing data among secondary school teachers and so those results are not informative.

25 While one might be tempted to conclude from this analysis that teachers in lower-income countries are relatively better off than those in higher-income countries, the poor living and working conditions in many low- and lower middle-income countries in our sample (not captured in our earnings data) may mean that even being paid several times the GDP per capita does not mean teachers enjoy the same quality of life as teachers in upper-middle- or high-income countries.

26 In their second jobs, teachers are mostly likely to work as small farmers, tutors, or vendors (Appendix Table A13, Table A14). See Appendix Table A15 for earnings of different teacher groups: teacher in public schools; primary school teachers; and secondary school teachers.

27 We also do this comparison by sector (Table A16, Table A17), using UN sector classification codes (United Nations Statistics Division, 2007). With secondary education, teachers earn more than workers in any sector except mining and financial services. With post-secondary education, teachers are much lower: post-secondary workers in agriculture, mining, manufacturing, construction, financial services, health and social services, public administration, and administrative services all earn more. Only workers in retail earn less.

28 If we include teachers and comparator workers with primary education in the regressions, then the teacher premium rises, which is unsurprising since non-teachers with only a primary school education make less.

29 Finan et al. (2017) document that public sector workers broadly are both paid more and are more likely to receive non-pecuniary benefits than other workers, across a sample of mostly low- and middle-income countries. Mizala and Ñopo (2016), who report that teachers earn less than other workers in Latin American countries, do not document non-pecuniary benefits.

30 In separate data on a wider range of benefits but asked only of teachers, we find that teachers – particularly civil servant teachers – across 10 African countries do report receiving a wide range of benefits (Table A22). For example, 54 percent of teachers in Benin (and 90 percent of civil service teachers) report receiving a housing allowance; those numbers are 67 percent (88 percent) in Côte d’Ivoire. The set of benefits vary across countries, but at least 30 percent of civil service teachers report at least three benefits in each of the 10 countries, and in one (Senegal) they report seven benefits.

## References

[b0005] Ahimbisibwe, P. (2019, May 21) No money for teachers’ salary raise – government*. The Daily Monitor.* Retrieved from https://www.monitor.co.ug/News/National/No-money-teachers--salary-raise-government/688334-5124224-1iat17/.

[b0010] Araujo M.C., Carneiro P., Cruz-Aguayo Y., Schady N. (2016). Teacher quality and learning outcomes in kindergarten. The Quarterly Journal of Economics.

[b0015] Barrera-Osorio F., Raju D. (2017). Teacher performance pay: Experimental evidence from Pakistan. Journal of Public Economics.

[b0020] Bashir S., Lockheed M., Ninan E., Tan J.P. (2018).

[b0025] Bold T., Filmer D., Martin G., Molina E., Stacy B., Rockmore C., Wane W. (2017). Enrollment without learning: Teacher effort, knowledge, and skill in primary schools in Africa. Journal of Economic Perspectives.

[b0030] Bold T., Kimenyi M., Mwabu G., Sandefur J. (2018). Experimental evidence on scaling up education reforms in Kenya. Journal of Public Economics.

[b0035] Bujakera, S. (2021, October 22). *School children storm Congo parliament over teacher strike*. Reuters. https://www.reuters.com/world/africa/school-children-storm-congo-parliament-over-teacher-strike-2021-10-21/.

[b0040] Chelwa G., Pellicer M., Maboshe M. (2019). Teacher pay and educational outcomes: Evidence from the rural hardship allowance in Zambia. South African Journal of Economics.

[b0045] Chetty R., Friedman J.N., Rockoff J.E. (2014). Measuring the impacts of teachers II: Teacher value-added and student outcomes in adulthood. American Economic Review.

[b0050] Cilliers J., Kasirye I., Leaver C., Serneels P., Zeitlin A. (2018). Pay for locally monitored performance? A welfare analysis for teacher attendance in Ugandan primary schools. Journal of Public Economics.

[b0055] Chudgar A. (2015). Association between contract teachers and student learning in five Francophone African countries. Comparative Education Review.

[b0060] Crawfurd L., Pugatch T. (2020). Teacher labor markets in developing countries. GLO Discussion Paper No. 473.

[b0065] Cruz-Aguayo Y., Ibarrarán P., Schady N. (2017). Do tests applied to teachers predict their effectiveness?. Economics Letters.

[b0070] Dal Bó E., Finan F., Rossi M.A. (2013). Strengthening State Capabilities: The Role of Financial Incentives in the Call to Public Service. The Quarterly Journal of Economics.

[b0075] da Conceição, L. (2017, September 12). Moçambique: Professores de Tete em greve por falta de salários. *DW*. Retrieved from https://www.dw.com/pt-002/mo%C3%A7ambique-professores-de-tete-em-greve-por-falta-de-sal%C3%A1rios/a-41723964.

[b0080] Das, J. (2017, July 21). Teachers’ salaries: Too many bucks for the bang? *Future Development at Brookings.* Retrieved from https://www.brookings.edu/blog/future-development/2017/07/21/teachers-salaries-too-many-bucks-for-the-bang/.

[b0085] de Ree J., Muralidharan K., Pradhan M., Rogers H. (2018). Double for nothing? The effect of unconditional teachers' salary increases on performance. Quarterly Journal of Economics.

[b0090] Dolton, P., Marcenaro-Gutierrez, O., Pistaferri, L., & Algan, Y. (2011). If you pay peanuts do you get monkeys? A cross-country analysis of teacher pay and pupil performance. *Economic Policy, 26*(65), 5, 7-55.

[b0095] Duflo E., Dupas P., Kremer M. (2015). School governance, teacher incentives, and pupil–teacher ratios: Experimental evidence from Kenyan primary schools. Journal of Public Economics.

[b0100] Elacqua, G., Hincapié, D., & Mariana Alfonso, E.V. (with Montalva, V., & Paredes, D.). (2018). *Profesión: Profesor en América Latina ¿Por qué se perdió el prestigio docente y cómo recuperarlo? Banco Interameri - cano de Desarrollo*. Retrieved from: https://publications.iadb.org/publications/spanish/document/Profesi%C3%B3n-Profesor-en-Am%C3%A9rica-Latina-Por-qu%C3%A9-se-perdi%C3%B3-el-prestigio-docente-y-c%C3%B3mo-recuperarlo.pdf.

[b0105] Evans D.K., Mendez Acosta A. (2021).

[b0110] Evans D.K., Mendez Acosta A. (2021). Education in Africa: What Are We Learning?. Journal of African Economies.

[b0115] Evans D.K., Yuan F. (2018). The working conditions of teachers in low- and middle-income countries. RISE Conference Paper.

[b0120] Ferraz C., Finan F. (2009). *Motivating Politicians: The Impacts of Monetary Incentives on Quality and Performance*. NBER Working Paper No. 14906.

[b0125] Finan F., Olken B.A., Pande R. (2017). The personnel economics of the developing state. In *Handbook of Economic Field Experiments*.

[b0130] Gilligan, D. O., Karachiwalla, N., Kasirye, I., Lucas, A. M., & Neal, D. (2022). Educator Incentives and Educational Triage in Rural Primary Schools. *Journal of Human Resources*. In Press.

[b0135] Goux D., Maurin E., Petrongolo B. (2014). Worktime regulations and spousal labor supply. American Economic Review.

[b0140] Gregory R.G., Borland J. (1999). Recent developments in public sector labor markets. Handbook of Labor Economics.

[b0145] Hanushek E.A., Piopiunik M., Wiederhold S. (2019). The Value of Smarter Teachers International Evidence on Teacher Cognitive Skills and Student Performance. Journal of Human Resources.

[b0150] Hanushek E.A., Rivkin S.G. (2006). Teacher quality. Handbook of the Economics of Education.

[b0155] Hanushek E.A., Rivkin S.G. (2010). Generalizations about using value-added measures of teacher quality. American Economic Review.

[b0160] International Labour Organization. (2012). *International Standard Classification of Occupations: ISCO-08*. Geneva: ILO. Retrieved from https://www.ilo.org/public/english/bureau/stat/isco/docs/publication08.pdf.

[b0165] International Labour Organization. (2020). ILOSTAT: The leading source of labour statistics. Retrieved from. https://ilostat.ilo.org/data/.

[b0170] Jackson C.K. (2012). Recruiting, retaining, and creating quality teachers. Nordic Economic Policy Review.

[b0175] Kontagora H.L., Watts M., Allsop T. (2018). The management of Nigerian primary school teachers. International Journal of Educational Development.

[b0180] Leaver C., Ozier O., Serneels P., Zeitlin A. (2021). Recruitment, effort, and retention effects of performance contracts for civil servants: Experimental evidence from Rwandan primary schools. American Economic Review.

[b0185] Mbiti I., Muralidharan K., Romero M., Schipper Y., Manda C., Rajani R. (2019). Inputs, incentives, and complementarities in education: Experimental evidence from Tanzania. The Quarterly Journal of Economics.

[b0190] Mincer J. (1974). Schooling, Experience, and Earnings. Human Behavior & Social Institutions No. 2.

[b0195] Mizala A., Ñopo H. (2016). Measuring the relative pay of school teachers in Latin America 1997–2007. International Journal of Educational Development.

[b0200] Motsoeli N. (2019).

[b0205] Muralidharan K., Sundararaman V. (2011). Teacher performance pay: Experimental evidence from India. Journal of Political Economy.

[b0210] Muralidharan K., Sundararaman V. (2013). Contract teachers: Experimental evidence from India. NBER Working Paper No. 19440.

[b0215] Mwenda D.B., Mgomezulu V.Y. (2018). Impact of Monetary Incentives on Teacher Retention in and Attraction to Rural Primary Schools: Case of the Rural Allowance in Salima District of Malawi. African Educational Research Journal.

[b0220] NCES. (2018). *Characteristics of Public School Teachers*. https://nces.ed.gov/programs/coe/indicator_clr.asp.

[b0225] New Zimbabwe. (2022, January 31). *Teachers vow not to return to class over poor salaries. New Zimbabwe.* https://www.newzimbabwe.com/teachers-vow-not-to-return-to-class-over-poor-salaries/

[b0230] Nyamai, F. (2021, February 15). *Knut threatens strike over stalled salary negotiations*. Nation. https://nation.africa/kenya/news/education/-knut-threatens-strike-over-stalled-salary-negotiations-3291340

[b0235] OECD. (2011). How much are teachers paid. *Education at a Glance.* Retrieved from http://www.oecd.org/education/skills-beyond-school/48631286.pdf.

[b0240] Okafor, O., Iriobe, C., Nwajiaku, C., & Onyene, V (2016). Service compensation and social prestige as predictive of Nigerian teachers' inclination to job retention. *Journal of Innovation in Education in Africa, 1*, 1 2016, 119-135.

[b0245] Programme d’analyse des systèmes éducatifs de la confemen (PASEC). (2015). PASEC 2014: Education System Performance in Francophone Sub-Saharan Africa. Retrieved from https://www.pasec.confemen.org/wp-content/uploads/2015/12/Rapport_Pasec2014_GB_webv2.pdf.

[b0250] Pugatch T., Schroeder E. (2014). Incentives for teacher relocation: Evidence from the Gambian hardship allowance. Economics of Education Review.

[b0255] Ugandan Ministry of Education and Sport and UNESCO IIEP Pôle de Dakar. (2014). *Teacher Issues in Uganda: A shared vision for an effective teachers policy.*

[b0260] United Nations Statistics Division. (2007). International Standard Industrial Classification of All Economic Activities Revision 4, Series M: Miscellaneous Statistical Papers, No. 4 Rev. 4, New York: United Nations. Retrieved from https://unstats.un.org/unsd/classifications/Family/Detail/27.

[b0265] UNESCO. (2009). *Universal primary education in Africa: the teacher challenge.* Retrived from https://unesdoc.unesco.org/ark:/48223/pf0000186643.

[b0270] UNESCO. (2020). *Institute for Statistics Education Statistics*. Retrieved from: http://data.uis.unesco.org/.

[b0275] UNESCO. (2016). *The World Needs Almost 69 Million New Teachers to Reach the 2030 Education Goals*. Retrieved from http://uis.unesco.org/sites/default/files/documents/fs39-the-world-needs-almost-69-million-new-teachers-to-reach-the-2030-education-goals-2016-en.pdf.

[b0280] U.S. Bureau of Labor Statistics. (2018). May 2018 National Occupational Employment and Wage Estimates United States. Retrieved from https://www.bls.gov/oes/current/oes_nat.htm#25-0000.

[b0285] U.S. Bureau of Labor Statistics. (2021). Labor Force Statistics from the Current Population Survey. Retrieved from https://www.bls.gov/cps/cpsaat18.htm.

[b0290] World Bank. (2018). *Learning: To Realize Education's Promise*. World Bank Group.

[b0295] World Bank. (2019a). World Bank Country and Lending Groups. https://datahelpdesk.worldbank.org/knowledgebase/articles/906519-world-bank-country-and-lending-groups.

[b0300] World Bank. (2019b). World Development Indicators. https://datacatalog.worldbank.org/dataset/world-development-indicators.

[b0305] World Bank. (2021). World Development Indicators. https://datacatalog.worldbank.org/dataset/world-development-indicators.

